# A Review of Self-Seeded Germanium Nanowires: Synthesis, Growth Mechanisms and Potential Applications

**DOI:** 10.3390/nano11082002

**Published:** 2021-08-04

**Authors:** Adrià Garcia-Gil, Subhajit Biswas, Justin D. Holmes

**Affiliations:** 1 School of Chemistry, Tyndall National Institute, University College Cork, T12 YN60 Cork, Ireland; chemistry@ucc.ie (A.G.-G.); j.holmes@ucc.ie (J.D.H.); 2 AMBER Centre, Environmental Research Institute, University College Cork, T23 XE10 Cork, Ireland

**Keywords:** nanowire, bottom-up synthesis, self-seeded growth, germanium, germanium alloys

## Abstract

Ge nanowires are playing a big role in the development of new functional microelectronic modules, such as gate-all-around field-effect transistor devices, on-chip lasers and photodetectors. The widely used three-phase bottom-up growth method utilising a foreign catalyst metal or metalloid is by far the most popular for Ge nanowire growth. However, to fully utilise the potential of Ge nanowires, it is important to explore and understand alternative and functional growth paradigms such as self-seeded nanowire growth, where nanowire growth is usually directed by the in situ-formed catalysts of the growth material, i.e., Ge in this case. Additionally, it is important to understand how the self-seeded nanowires can benefit the device application of nanomaterials as the additional metal seeding can influence electron and phonon transport, and the electronic band structure in the nanomaterials. Here, we review recent advances in the growth and application of self-seeded Ge and Ge-based binary alloy (GeSn) nanowires. Different fabrication methods for growing self-seeded Ge nanowires are delineated and correlated with metal seeded growth. This review also highlights the requirement and advantage of self-seeded growth approach for Ge nanomaterials in the potential applications in energy storage and nanoelectronic devices.

## 1. Introduction

The discovery of the semiconducting behaviour in materials, by Alessandro Volta in the 18th century, settled an inconceivable breakthrough in the technological progress that was going to be developed in the next centuries [1]. Later on, in the years that followed the visionary work of Wagner and Ellis in the 1960s [2], several semiconductor compounds were successfully synthesised in nanostructure form, including the widely popular Si and Ge structures [3,4]. As stated by Moore’s Law [5], the semiconductor industry had no choice but to follow a continuous drive towards device miniaturisation for a greater integration in electronic devices. The miniaturisation drive in the semiconductor industry has been used as a guide for long-term planning and also to set targets for research and development in other areas such as energy harvesting, energy storage, photonics, sensing, and catalysis. Thanks to developments in nanoscale materials and devices, we are now embarking on a nanotechnology time in history. The *leitmotiv* which powers the investigation of nanoscale materials is the captivating new chemical and physical properties exhibited when compared to their analogous bulk material [6]. Particularly, some of the significantly size-dependent features are the physical, electrical and optical properties [7,8,9]. These materials are gathered in a range of structures such as 0D (quantum dots) [10], 1D (nanorods [11], nanotubes [12], nanobelts [13] and nanowires [14]), 2D (nanofilms [15], nanodisks [16], nanoplates [17] and nanosheets [18]) and 3D (assemblies of nanocrystals [19] and nanocomposites [20]).

The revolution of one-dimensional (1D) nanomaterials started with Haraguchi et al. [21], who achieved the first device fabricated from 1D nanostructures, back in 1992. The semiconductor nanowires of small diameters make them attractive for potential quantum confinement effects, while their unconstrained lengths make it easy to integrate them into already existing devices. This escalated the interest in 1D nanostructures for all kinds of potential applications (photocatalysts [22], magnetic storage medium [23], nanoscale sensors [24], optical devices [25], solar cells [26], liquid crystal devices [27], etc.). In particular, research into group IV semiconductor nanowires has been of high interest since the beginning of the 2000s [28]. Within group IV elements, silicon has been the material of choice for the microelectronics industry due to its abundancy on the Earth’s crust (and cost-effective as a consequence), non-toxic nature and chemically stability [29]. As well, it presents high carrier mobility and optimum structural and electrical characteristics of the Si/SiO_2_ interface, all advantageous features for different electronic, optoelectronic, and energy applications. Thus, a key requirement is to research into materials compatible with well-developed Si-based industrial technology. Although the first transistor was made using Ge in the 1940s [30,31], its lower natural abundance (and higher price as a consequence) and less stable external oxide layer forced the industry to replace it with Si [32]. However, the current development of high-*k* dielectric materials has boosted the research on synthetic protocols applied to Ge nanostructure formation, and nanowires in particular [33,34].

Germanium exhibits superior features to Si such as superior charge carrier transport due to the lower effective mass of electrons and holes [35], and presents quantum confinement effects at larger dimensions (Bohr radius of 24.3 nm and 4.9 nm for Ge and Si, respectively) [36,37,38,39]. Electron and hole mobility in bulk germanium at room temperature is 2.5- and 3.5-fold higher than Si, respectively. These characteristics suggest that Ge could be utilised in the fabrication of high-performance transistors with nanoscale gate lengths, especially for faster-switching and higher-frequency devices. The greater electrical conductivity of Ge along with higher lithium diffusivity also make it an ideal candidate in niche energy storage applications such as small high-tech devices. Additionally, germanium displays a smaller indirect band gap of 0.66 eV (at 300 K) with a very small difference with the direct bandgap (~140 meV) compared to Si (indirect band gap of 1.11 eV with the large direct–indirect bandgap difference of 2.4 eV at 300 K). This narrow difference between direct and indirect band gap implies the possibility of obtaining a direct band gap in Ge by the application of external strain or alloying, ideal for optoelectronic and photonic devices. Fortunately, the similarities in crystal structure between Si and Ge simplified the development of bottom-up synthetic techniques and catalytic growth procedures to obtain high-quality Ge nanostructures, and nanowires in particular [40]. This synthetic progress has been useful not only in nanoelectronic devices [41,42,43], but also in other applications fields such as solar energy conversion [44,45], memory applications [46], biological nanosensors [47,48], catalysis [49], nanoelectromechanical systems [50] and energy storage [51,52]. However, Ge possesses an unstable, non-uniform oxide surface on both bulk and nanowire surfaces which gives rise to a poor Ge/GeO_x_ interface characterised by a high density of surface states, limiting its implementation in electronic devices. However, as mentioned before, this is now compensated by the development of high-*k* dielectrics.

Ge nanowires have been produced using a wide variety of synthetic protocols, most of which are based on the vapour–liquid–solid (VLS) or VLS-like mechanisms [53,54,55]. These approaches are based on a three-phase system (growth medium, seed particle and nanowire/substrate support) where nanowire growth occurs. The nature of the selected nucleation seed (external or self-seeded) plays a crucial role in nanowire growth development. However, even though some external catalyst seeds may present synthetic advantages, they might also diffuse and incorporate into the structure of nanowires. This phenomenon might lead to impurity incorporation into the nanowire structure and a significant modification of the semiconducting properties of the nanowires. Catalyst-free and self-seeded growth approaches have demonstrated to be able to obtain competitive nanowire growth rates and narrow diameter distribution without affecting the chemical and physical nature of the resultant nanomaterial.

Among all applications for self-seeded Ge nanowires, energy storage, lithium-ion batteries (LIBs) in particular, have garnered much interest [56,57]. Semiconductor nanowires are very attractive for LIBs due to the good electronic conduction, the short lithium-ion diffusion distance, and the high surface contact area with battery electrolytes [56,58]. LIBs have been accepted as the pre-eminent energy storage technology for portable electronic devices due to several presented advantages such as high energy/power density, long life and eco-friendly features [59,60]. However, state-of-the-art LIBs still do not meet the demands of high-performance requirements in the current market, i.e., electric vehicles, and germanium is one of the most promising candidates for next-generation LIBs. As mentioned before, features of germanium such as the theoretical specific capacity of 1624 mA h g^−1^ (for Li_22_Ge_5_), the high electrical conductivity (10^4^-fold higher than that of Si), the high Li-ion diffusivity (400-fold greater than that of Si) and a volumetric capacity of 7366 Ah L^−1^ place germanium (and its alloys or composites) in a privileged position to replace common graphite as a future anode for LIBs [56,61,62,63].

Enormous interest in germanium one-dimensional (1D) materials is apparent from the fact that several review articles, including articles from our research group, were published on this subject in recent years [64,65,66,67]. These reviews primarily cover the three-phase bottom-up growth of Ge nanowires. However, none of these reviews emphasise the critical aspect of unwanted impurity incorporation from the catalyst in a conventional VLS-like growth. These unwanted impurities located in the conduction band can impair nano/optoelectronic device performance by acting as scattering centres. Impurity incorporation from the metallic seeds into the nanowire structure can also affect the mechanical (along with the electrical) properties of germanium, and potentially the capacity of energy storage devices such as Li-ion cells. Additionally, nanowire growth using external metal seeds could add to the expense of the synthetic process. Thus, generating a knowledge base on the non-catalytic (seedless and self-seeded) growth of germanium is essential for its implementation in functional devices. In this review, we will evaluate synthetic methods and growth mechanisms for self-seeded Ge nanowire growth and the current state of the art in seedless Ge nanowire materials. First, we will introduce the general growth mechanisms for both externally seeded and self-seeded Ge nanowire growth and a comparison between them. The we will explore different vapour and solution-based synthetic methods employed to grow Ge nanowires with a focus on self-seeded bottom-up approaches. We will also discuss the growth mechanisms behind the growth of Ge nanowires via a self-seeded approach. Finally, we will focus on the functionality of self-seeded Ge nanowires and their applicability in devices, in particular as anode materials in lithium-ion batteries.

## 2. Germanium Nanowire Growth Mechanisms

### 2.1. Primary Growth Paradigms for Nanowire Growth

Ge nanowires can be synthesised using a wide variety of synthetic protocols, most of which are based on the three-phase vapour–liquid–solid (VLS) mechanism and its derivatives such as vapour–solid–solid (VSS), solution–liquid–solid (SLS) or supercritical fluid–solution–solid (SFLS) growth paradigms. Less popular growth mechanisms, such as vapour–solid (VS) and oxide-assisted growth (OAG), have also been explored for Ge nanowire growth. Although other growth mechanisms have also been used for nanowire synthesis such as solvent–vapour, epitaxial growth or solid–liquid–solid, this section will only focus on the most used growth mechanisms for Ge nanowires, which are also relevant in understanding the seedless and self-seeded growth mechanisms of nanowires. A brief discussion on the different bottom-up growth mechanisms involving seed/catalyst, e.g., VLS, VSS) or no participation of seed, e.g., VS, OAG, is presented in this section.

Generally, the names attributed to growth mechanisms tend to be quite self-explanatory and are derived from the physical states of the phases involved. Some of the growth methods, e.g., VLS, VSS, SLS, and SFLS, differ only in the reaction medium, but the growth mechanism is otherwise identical. The initial word, such as “vapour”, “solution” or “supercritical fluid” refers to the medium where the reaction is going to take place and the source of the reaction material. The second word, “liquid” or “solid”, indicates the state of the catalyst seed or droplet at the reaction temperature. The last word makes reference to the substrate and the single-crystal nanowires formed, where the growth takes place. Successful implementation of these growth mechanisms requires the precursor species to be delivered and reach a catalyst droplet (liquid or solid), where supersaturation and nucleation, and finally crystallisation into a solid nanowire structure will take place. A generalisation for the different growth mechanisms is that the growth rate at the seed-crystal solid interface is faster than the growth rate at any of the other interfaces. This point will be discussed in detail in later sections.

While the catalyst for the VLS growth of Ge nanowires is in the liquid state, the catalyst for VSS growth is at a temperature below the melting point, in the solid state. The state of the catalyst is assumed to be at a temperature below its melting point (or eutectic point of the mixture) based on bulk phase diagrams [68]. However, at the nanoscale, a modification in the phase diagram (or melting point of the nanoscale seed) may occur and a shift of the metal-semiconductor eutectic towards lower temperature may lead to attribution to a wrong growth mechanism [69,70,71]. Likewise, the eutectic point shift might differ between materials with the same-sized nanoparticles [72,73]. The state of the seed can also influence nanowire growth. For example, a reduction in the growth rate of nanowires via VSS growth by an order of magnitude compared to the VLS growth under identical growth conditions has been identified [74]. This is attributed to weaker surface reactivity (lower eutectic point) and lower diffusivity through the solid droplet compared to the growth with the liquid nanoparticle.

Solution-based (SLS [75] and SFLS [76]) synthesis mechanisms are analogous variants of the VLS mechanism, although conducted in solution or solvent dispersions. Solution-based mechanisms are a promising approach for the mass production of nanomaterials with excellent controls of the composition and morphology. They are usually conducted in the presence of coordinating solvents, which help in tuning the growth kinetics and passivating the generated structures. These mechanisms present some clear advantages over other widely used mechanisms. Narrow size distributions (diameter as small as 5 nm) and the crystallinity could be achieved at much lower growth temperatures (usually 200–350 °C) compared to vapour-phase growth [76,77]. Better control over nanowire morphology and crystallinity was achieved via a flow-through solution-based process compared to a batch growth process [76,77,78]. Nevertheless, a clear challenge that still exists is the significant amount of carbonaceous by-products formed during the reaction, which often limits reaction yields and contaminates the resulting nanowires, although this issue has been used as an advantage for battery applications and will be discussed later.

A SFLS growth mechanism in particular is achieved when the solvent is at a temperature and pressure beyond its critical point. Above the critical point, the vapour and liquid coexist and a single-phase fluid with intermediate properties is generated which presents a unique density, low viscosity, high diffusivity and low surface tension [79]. These particular properties allow significant synthetic tunability by varying temperature and/or pressure, acquiring desired gas-like or liquid-like conditions. Growth under these conditions provides: (i) higher chemical flexibility; (ii) dispersion of bigger nanoparticle seeds; (iii) precursor solubilisation at higher concentrations than usual and (iv) growth under high temperatures in a solution phase by increasing the boiling point of the solvent. The yield of the nanowires from SFLS growth is surprisingly high, extended up to milligram quantities of different single-crystal nanowires [80,81,82]. The main flaws of the SFLS growth are the possible nanoparticle seed agglomeration and coarsening leading to broad nanowire diameter distributions [79]. Additionally, nanowire nucleation and growth take place at a higher rate than VLS growth (μm/min in SFLS while nm/min in VLS), which is often too fast for quantitative analysis of growth kinetics and mechanics. At the same time, sequential introduction of precursors or concentration adjustments are much more limited, which restricts the synthesis of axial heterostructures [83].

The vapour–solid (VS) growth mechanism is one of the most widely employed mechanisms for the general growth of nanomaterials. This mechanism is a catalyst-free (unseeded) mechanism, which means it does not require the presence of a third party to act as a nucleation point. Instead, VS growth only involves two phases and growth occurs directly from the vapour-phase source species condensing to the crystalline solid-phase nanostructure. The required growth temperature for this mechanism is higher than in other catalytic methods due to the absence of an energetically favoured catalytic point (the seed). This mechanism is mainly governed by the temperature and pressure of the reaction and the nature of the surface where the deposition takes place. The main advantage of this method is the absence of any contamination resulting from parts of the seed becoming trapped in the crystalline structure of the nanowires. Ge nanowires produced via the VS mechanism are generally free from stacking faults and metal contamination and can be more efficient light emitters than the seeded nanowires, e.g. Ni seeded nanowires [84,85].

Oxide-assisted growth (OAG) is another mechanism employing high temperature as the main driving force for nanowire growth. In this process, instead of a metal or metalloid catalyst seed, oxides play a key role in the nucleation and growth of the nanowires. Nanowires with preferential growth directions, uniform diameters, high yields and high-purity can be obtained through OAG [86]. While nanowire diameter is limited by the seed diameter in VLS-like growth, in the OAG, a very narrow diameter (even ~1 nm) can be achieved by careful control of gas flow and substrate temperature [87]. Another important advantage is the use of simple elemental and oxide powders as precursors, avoiding poisonous and flammable precursor gases such as silane or germane. However, the main drawbacks are the high reaction temperature required (enough to vaporise the precursors under vacuum) and the unavoidable oxide shell growth, which must be removed before implementation in electrical or optical devices. In a conventional OAG process, the first step consists of a thermal evaporation of a mixture of the material and its oxide. Despite the high temperatures used, most of the solid source materials may not sublime in a regular atmosphere, thus high-vacuum conditions are required. Reactive oxide clusters (a mixture of Ge and GeO_x_) are also formed and deposited while generating some bonding with the substrate itself and limiting the mobility across the surface. As a result, non-bonded and highly reactive atoms present in the cluster are exposed to the vapour phase. Oxides play an important role here with the formation of intermediate catalyst-seed-like clusters [88,89,90]. Initial clusters may act as nuclei for absorption of additional clusters or other vapour species, serving as a driving force for nanowire growth [91,92]. In this process, oxygen atoms are desorbed from the clusters during crystallisation, inducing the diffusion of the oxygen to the surface and producing a chemically inert oxide sheath [93].

### 2.2. Why Self-Seeded Growth? Disadvantages of Catalytic Ge Nanowire Growth

As pioneered by Wagner and Ellis [2], metal catalyst-based techniques have become the most commonly employed for growing 1D semiconductor nanostructures. The catalytic particle assisting the growth becomes the crucial point of the overall growth process. This is not only because the size of the particle determines the diameter of the nanowires, but also the catalyst position on the substrate may dictate nanowire placement [94,95]. Seeded growth involving a metal catalyst is based on the idea that the energy barrier required for a given material to nucleate heterogeneously onto a pre-existing seed is lower than the necessary activation energy to induce, homogeneously, self-nucleation.

#### Different Catalyst Type and Their Effect on Physical Properties of Nanowires

Au is the most extensively studied catalyst for Ge nanowire growth. Some of the main reasons for it to be considered the standard catalyst are its low toxicity, chemical stability when exposed to air, low vapour pressure at high temperatures and simple processing. Although Au forms a low-temperature eutectic with Ge (361 °C) [96], there are certain disadvantages associated with using the metal, which led to the screening of other elements and alloys as catalytic seeds for Ge nanowire growth. The catalysts used for Ge nanowire growth are generally categorised into three groups [97]: (i) type A, i.e., Au, Ag and Al, with simple binary phase diagrams and high eutectic solubility (>10% of Ge in the eutectic alloy); (ii) type B, i.e., In, Sn and Bi, with also simple binary phase diagrams but low eutectic solubility (typically <1% of Ge in the eutectic alloy); and (iii) type C, i.e., Fe, Ni and Cu, germanide-forming catalysts which result in complex binary phase diagrams. Due to the high melting point (>1000 °C), the type C catalyst will generally not be in a liquid state but in a solid state to induce VSS growth [98].

Within type A catalysts, Au, which is an expensive and rare metal, emerges as the predominant catalyst for Ge nanowires, as other type-A metals (Ag or Al) form relatively high-temperature eutectics with a high Ge solubility. However, with all metal catalyst particles used for nanowire growth, the effect of possible metal incorporation into the crystal structure of nanowire can become a critical point for electronic transport. The high diffusion constant of many metals in Ge makes impurity incorporation likely to occur during growth [99]. A significant modification of semiconducting properties, e.g., band gap, is produced when the incorporation of 10^−7^ catalyst atoms in the nanowire is surpassed [100,101]. The presence of these impurities can not only introduce deep defect levels in the semiconductor band gap, making it unfavourable for electronic applications, but also negatively affect energy storage applications by inducing irreversible capacity losses in anodes for Li-ion batteries [102], although most deep-level recombination processes and scattering occur from nanowire surface states, which may not be altered by the presence of Au in the bulk of the nanowires [103,104]. This is of particular importance for Ge, which display higher surface-state densities than Si (10^14^ and 10^12^ cm^−2^, respectively) [105]. Selective chemical etching, e.g., in aqua-regia, has been occasionally used to remove Au present on the surfaces of nanowires, but Au atoms trapped into the nanowire structure during growth might still affect the properties of these nanowires [106,107].

All these concerns over the use of Au catalysts fuelled the investigation of more suitable catalyst alternatives for the synthesis of semiconductor nanowires, Ge nanowires in particular [108]. Ag, as well as Ag/Au alloys, have shown high efficiency in the growth of Ge nanowires [109]. Al is also presented as one of the critical seed-metals as it creates low-level traps in the Ge nanowire structure [110] and also acts as a p-type dopant [111]. However, the sensitivity and high reactivity of Al with oxygen limit its usage in most synthetic setups, requiring demanding oxide removal steps. For type-B catalysts, the diffusion of the seed into the nanowire structure might be seen as an advantage since the presence of those elements can act as dopants. However, the solubility of Ge in these metals or metalloids is usually low (<1%), which might lead to meta-stable structures when high incorporation ratios are achieved [112]. Zn is the least interesting one within this group because it can introduce electron traps (such as type A catalysts). Nevertheless, good-quality nanowires have been obtained with Zn [113]. Cd and Tl are not very suitable for Ge nanowire growth due to droplet–nanowire system surface tension, a crucial element in nanowire growth. Group III, i.e., Ga [114] and In [115], IV, i.e., Sn [116] and Pb [117] and V, i.e., Sb [40] and Bi [118], elements are much more interesting as seed materials due to their low eutectic temperatures while having potential doping capacities as both n-type and p-type dopants. Type C catalysts, e.g., Cu [119] and Ni [120], are of particular interest due to their compatibility with group IV materials, and thus, potential integration in the Complementary Metal–Oxide–Semiconductor (CMOS) industry [121]. As type B catalysts are promising due to their doping capacity, type C materials have become popular due to their promising ability to be used in microelectronic applications where properties such as high conductivity and high-quality crystal structures are required. However, oxidation of these catalysts could be an issue along with the slow nanowire growth rate and germanide phase formation, which may limit their application.

## 3. Self-Seeded Germanium Nanowire Synthesis Methods

Ge nanowires are commonly fabricated by either top-down or bottom-up processes. Each of the approaches presents intrinsic advantages and limitations and may be adopted under the appropriate circumstances. In the semiconductor industry, since its onset in 1959, top-down approaches, the optical lithography or photolithography, was utilised to fabricate nanowires [122]. Photolithography involves several steps, comprising material removal from bulk samples through local etching and deposition processes, to build up a nanowire structure. This technique has been able to design and produce nanowires with dimensions down to the 10 nm range [123]. Top-down methods also include electron beam and focused ion-beam lithography, both less present at the industrial-scale production. The main drawback presented by photolithography is the surface roughness of nanowires generated which may result in considerable diameter variability. Rough surfaces are also susceptible to increase surface scattering. Additionally, top-down processing also has a limitation in achieving engineered nanostructures such as heterostructure and alloys.

In contrast, bottom-up techniques present a high degree of freedom in combining materials to construct very complex structures through self-assembly. After the first demonstration of bottom-up nanowire synthesis in 1964 [2], this method became the most common path to obtain any anisotropic growth at the nanoscale. A wide range of bottom-up approaches have been developed, and most of them are well known for the formation of high yields of small-diameter nanowires, while maintaining low costs of production. However, usage of external catalysts for nanowire growth leads to metal incorporation in the nanowire atomic structure, which critically alters the semiconducting properties of the nanostructures (discussed in detail in Section 2.2). Another potential drawback for bottom-up growth is the precise placement with high regularity over large surface areas, as required for integrated circuits. This section will review the synthetic methods widely used for Ge nanowire growth, with a special focus on self-seeded approaches.

### 3.1. Bottom-Up Synthesis of Self-Seeded Germanium Nanowires

Bottom-up techniques have the potential to outperform the limits and functionalities of top-down strategies controlling morphology, composition and structure within a narrow distribution range. Within bottom-up synthetic approaches, different explored routes can be classified as: (i) vapour-phase growth, (ii) solution-based growth and (iii) growth techniques using templates.

The standard process for both vapour-phase and solution-based techniques can be described by three simple steps. Initially, a gaseous Ge precursor (originally gas or volatilised liquid) is introduced into the system. An inert carrier gas such as Ar is employed to transport the precursor in the reaction chamber, providing an oxygen free environment. The supplied gaseous precursor (either in molecular or atomic form) gathers around the nanoparticle seed (either in solid or liquid phase) deposited over a substrate. This is followed by the decomposition of the precursor molecules on the surface of the nanoparticle seed, as well as adsorption and diffusion of the Ge atoms into the nanoparticle seed. Finally, when supersaturation is reached in the seed particle, a preferential precipitation occurs as a single-direction elongation (usually at the interface between the seed and the substrate) to form one-dimensional structures. Most often, this deposition process is thermally driven, but several other perturbation processes (growth enhancement by laser [124], X-ray [125], microwave [126]) have also been described.

The three-phase bottom-up Ge nanowire growth may also be achieved without the use of an external catalyst. Ge itself plays an important role in the formation and growth of Ge nanowires via a self-seeded mechanism in a VLS paradigm. This is usually achieved when in situ Ge nucleation seeds form, requiring no pre-deposited nanoparticle seeds or films of Ge, generating nanowires. More detailed analysis of the self-seeded growth of Ge nanowire will be discussed in Section 4.

#### 3.1.1. Vapour-Phase Growth of Self-Seeded Nanowires

Within the vapour-phase growth group of techniques, chemical vapour deposition (CVD) and physical vapour deposition (PVD) can be distinguished. While PVD is based on physical mechanisms to deposit nanomaterials, CVD relies on chemical reactions to obtain different nanostructures.

##### PVD Growth of Self-Seeded Nanowires

In PVD growth, a solid precursor transforms into vapour using a laser or high-power electrical tools. Once transferred into the reaction chamber, the vaporised material is adsorbed on the substrate or nanoparticles yielding to the growth of desired material, thus not relying on any chemical process. This growth method has advantages not only as eco-friendly technique but also when growing extremely hard and corrosion-resistant materials because of their high-temperature laser-ablation resistance. However, the main flaw for its scalability is the high cost due to the slowness of the process and the intense heating and cooling procedures required.

**Figure 1:**
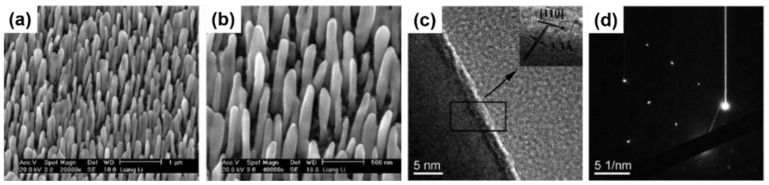
(**a**,**b**) Scanning electron microscopy (SEM) images of the Ge nanostructures deposited at a substrate temperature of 330 °C with a flux angle of 87° and a deposition rate of between 0.1 and 0.2 Å s^−1^. Scale bar of (**a**): 1 μm and the magnified image of (**b**) the scale bar is: 500 nm. (**c**,**d**) HRTEM images and SAED patterns of Ge nanowires fabricated at a temperature of 330 °C. Reprinted with permission from ref. [134]. Copyright 2008 Wiley Online Library.

Oblique angle deposition (OAD), utilising the epitaxy of a growth substrate is one of the simplest examples of PVD. Epitaxial growth refers to atoms nucleated and grown on a single-crystal face ordered in a relative orientation to a substrate. This technique bases its capacity of growing nanowires only on the appropriate selection of a substrate crystal orientation, the temperature and precursor evaporation rate, and the control of the deposition angles [127]. Taking advantage of the possible crystallographic correlation between the single-crystal substrate and the crystal structure of the nanowires (lattice matching), a predominant growth orientation is achieved [35]. Nanowire growth can also be produced when there is a large lattice mismatch between a nanowire and substrate material [128]. The relative substrate-deposit orientation depends on the structure of the crystal planes in contact and the atomic interactions across the interface, although, a mask layer, i.e., SiO_x_, is often used to allow growth on specific areas of a substrate [129]. Growth takes place at a very slow rate on low-surface-energy side facets, as no catalyst seeds are used and the process is only controlled by temperature, which significantly limits its tunability [130,131]. However, a drawback of this technique is the formation of kinked nanowires [132,133]. Electron beam melting (EBM) is an analogous technique based on the use of suitable substrates to control the alignment and crystal orientation of nanowires. A detailed study about self-seeded Ge nanowire growth by OAD was published by Li et al. [134], the first and only attempt to grow pure Ge nanowires by OAD. Different substrate temperatures were screened, under an optimised flux angle and deposition rate variables, and long and self-standing nanowires were obtained at a temperature of approximately 330 °C. As the nanowires did not display a straight morphology with smooth surfaces (see SEM images in Figure 1a,b), details on mean diameter, diameter variation and length of the nanowires were not disclosed. The crystallinity of the nanowires was confirmed by Raman spectroscopy and X-ray diffraction (XRD), obtaining the highest crystallinity for samples grown at a high temperature (330 °C). Transmission Electron Microscopy (TEM) (see Figure 1c,d) further validated the presence of amorphous Ge at 130 °C, polycrystalline Ge at 230 °C and highly crystalline structures growing in the (110) direction at a temperature of 330 °C. Thermal stability tests were also performed, presenting Ge nanowires with excellent structural cohesion below 500 °C. At 500 °C nanowires started to melt and aggregate together, and above 580 °C they were all melted and aggregated as particles.

##### CVD Growth of Nanostructures

In CVD growth, a vapour-phase chemical reaction takes place close to the surface of the substrate, which is maintained at the growth temperature during the whole deposition period. Depending on the processing conditions, CVD can be classified as metal-organic CVD, low-pressure CVD, plasma-assisted CVD, hot-wall CVD, etc. As a widely studied technique, and probably the most popular for nanowire growth, CVD allows the formation of a variety of nanomaterials from a vast number of material sources. CVD is based on a near-equilibrium process, which grants a firm control over composition, dimension, location, and morphology of the desired nanomaterial, as well as the impurity concentration and distribution (in situ doping). CVD as a method for growing semiconductor nanowires has advantages or disadvantages that mainly depend on the reaction temperature used. For example, working at low temperatures (<600 °C) makes CVD more compatible with Si processing, so any innovation is more likely to be used for industrial applications [135]. At temperatures <600 °C, narrow and uniform diameter nanowires, along with highly-crystal and well faceted structures are achievable. In situ doping processes are also more readily accomplished, allowing facile tuning of nanowire properties [136,137]. However, there is a wider choice of precursors when working at high temperatures (>700 °C), which also allows a greater degree of freedom to vary growth conditions (temperature and pressure). The main concern with using high temperatures is the aggregation and Ostwald ripening processes suffered by the nanoparticle seeds due to the surface diffusion. These effects result in large seed particles formation, which inhibits the formation of uniform diameter distributions. In this regard, self-seeded bottom-up grown carbon-capped seeds formed in situ has an advantage over the common metal-assisted CVD growth via VLS technique. The main drawbacks of the popular CVD technique are the numerous control variables to consider when designing an experiment and the use of harmful gases and/or precursors.

##### Self-Seeded Nanowire Growth by CVD

**Figure 2:**
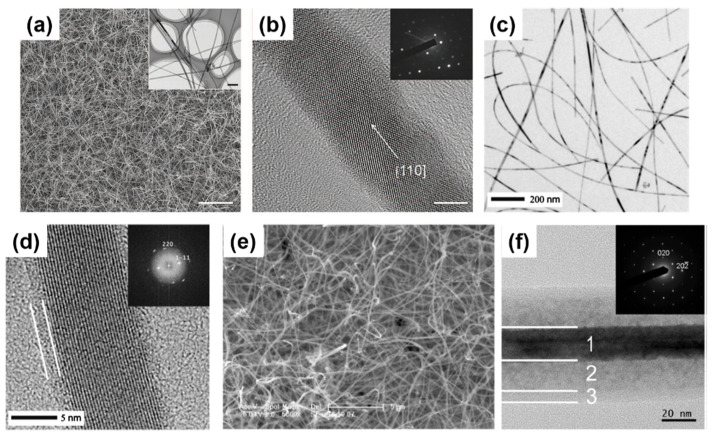
(**a**) A SEM image showing high-density, uniform Ge nanowires grown from a planar thin SiO_x_ film on a Si surface; scale bar: 3 μm. Inset: a TEM image of the Ge nanowires, scale bar: 100 nm. (**b**) A lattice-resolved TEM image of a single-crystal Ge nanowire. The arrow highlights the nanowire axis, which corresponds to the <110> direction; scale bar: 5 nm. Inset: the corresponding diffraction pattern. (Parts (**a**,**b**) are reprinted with permission from ref. [138]. Copyright 2009 American Chemical Society). (**c**) Bright-field TEM image of Ge nanowires prepared on a stainless steel AISI 310 substrate. (**d**) HRTEM observations of Ge nanowire elongated along the [110] direction prepared on a stainless steel AISI 310 substrate (the amorphous germanium oxide shell is indicated). (Parts (**c**,**d**) are reprinted with permission from ref. [141]. Copyright 2010 IOP Publishing). (**e**) SEM observations of the sample annealed at 700 °C. (**f**) HRTEM observations of a single Ge nanowire from a sample annealed at 700 °C. Nanowire elongated along the (110) plane, electron diffraction of Ge along [101] as an inset (1-core, 2-inner jacket, 3-outer jacket). (Parts (**e**,**f**) are reprinted with permission from ref. [142]. Copyright 2009 IOP Publishing).

Kim et al. [138] used a hydrogen-terminated patterned Si substrate surface as a reactive surface to accommodate the growth of Ge nanowires in a common CVD setup. The use of the GeH_4_ precursor mixed with H_2_ gases yielded highly dense growth of nanowires (see Figure 2a,b) with aspect ratios above 10^3^ [138]. The same precursor was utilised to evaluate the effect of X-rays in Ge nanowire growth in a CVD reaction [125]. Ge nanowire with high aspect ratios, with lengths up to 20 μm, and a high degree of kinking were produced at a low synthesis temperature of 300 °C. Aggregated spheroids or amorphous hydrogenated Ge were not found in the samples [139,140], and the nanowires obtained were highly kinked. Broad diffraction peaks determined by X-ray Diffraction (XRD) pointed towards low crystallinity in the nanowire samples, further confirmed by the TEM observation of a small yield of amorphous Ge(a-Ge) phase. Dřínek et al. [141] utilised alternative commercial precursors such as (GeMe_3_)_2_ for the Ge nanowire growth without the presence of any additional catalysts. A growth temperature of 490 °C lead to the formation of high-quality Ge nanowires on substrates such as Ta, SiO*_x_* and W. While kinking was absent in the nanowires (see Figure 2c) bending of the nanowires was observed (see Figure 2d) which may inhibit their application in electronic/optoelectronic devices. The same authors experimented with other Ge precursors, such as (SiMe_3_)_3_GeH, in a single-step CVD approach which created unusual nanowire morphologies, such as core-shell Ge-Si/C nanowires (see Figure 2e) [142]. A Ge core with a variable diameter between 5 and 40 nm was coated by a 2-layer shell of Si and C (see Figure 2f) with a total thickness of 5 to 20 nm. The shell part was constituted by an inner coating of 5 nm Ge crystallites in an amorphous body, surrounded by a completely amorphous outer coating. These Ge nanowires with an outer diameter up to 100 nm (core and shell) generally presented a crystalline Ge core growing along the [110] direction, exhibiting single-crystal Ge structure without any defect [142].

**Figure 3:**
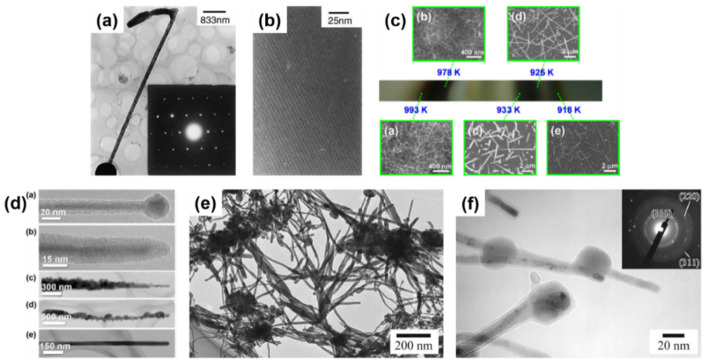
(**a**,**b**) TEM image and a SAED pattern (inset) along the (101) direction and a lattice fringe image of single-crystal Ge (high-pressure phase) nanorods. (Reprinted with permission from ref. [146]. Copyright 2002 Elsevier B.V). (**c**) Optical micrographs of an as-synthesised nanowire specimens grown on a Si substrate; the corresponding temperatures and SEM images of the as-synthesised nanostructures in different regions. (**d**) TEM images, corresponding to (**c**), of Ge nanostructures from (**a**–**e**), respectively. ((**c**,**d**) are reprinted with permission from ref. [149]. Copyright 2010 IOP Publishing). (**e**) TEM image of deposits obtained at a Ge content of 40 at.% and an Ar pressure of 0.1 MPa and (**f**) TEM image of nanowires and their tip parts in deposits, and inset: corresponding SAED pattern. (Reprinted with permission from ref. [151]. Copyright 2017 Scientific Research Publishing).

CVD (as well as PVD) processes can accommodate thermal evaporation of solid Ge precursors for Ge nanowire growth; a simple and scalable process which does not require hazardous or toxic precursors but a powder or solid pellets. Typically, the thermal evaporation technique requires the use of very high temperature (>938 °C, corresponding to Ge melting point), although the evaporation temperature can be lowered (~700 °C) with the use of metal catalyst seeds [37,143,144,145]. Wu et al. [146] first attempt to grow self-seeded Ge nanowires by the thermal evaporation of Ge powder at a temperature of 1050 °C, under a flowing Ar/H_2_ atmosphere for 2.5 h. Very poor-quality Ge nanorods (see Figure 3a), with diameters ranging from between 20 and 200 nm and lengths up to 5 μm, were obtained with a mixture of crystalline and amorphous structure (see Figure 3b). However, the nanorods produced showed a mixture of tetragonal, cubic and amorphous Ge structures, including a combination of these morphologies in single nanorods. GeO_2_ has also been used as an alternative solid precursor, as it can be reduced in situ by H_2_ at high reaction temperatures [147,148]. The crystallinity of the Ge nanowires produced can be tuned to be either amorphous, polycrystalline or single crystalline, by modifying the substrate temperature and/or evaporation rate. Wu et al. [149] used the GeO_2_ precursor to synthesise self-seeded Ge nanostructures under H_2_ flow at a temperature of 1100 °C (727–627 °C in the deposition zone). Different 1D structure morphologies (such as straight nanowires, sphere-capped nanowires and tapered nanowires) were obtained along the substrate length, each corresponding to a different growth temperature (see Figure 3c). Uniform nanowire morphology was obtained at the coolest deposition area of the substrate (645 °C) where Ge nanowires with diameters ranging between 20 and 120 nm, lengths between 0.5 and 10 μm, and smooth surfaces and very few structural defects were produced (see Figure 3d). An amorphous shell of between 1 and 7 nm in thickness was also observed on the surfaces of these nanowires. Other research groups also utilised GeO_2_ as a precursor for growing Ge nanowires, combined with H_2_ and various hydrocarbon molecules, e.g., methane, acetylene and ethanol, to achieve self-seeded core-shell 1D nanostructures (shells composed of carbon) [147,148,150]. These carbon-based self-seeded Ge nanowires were shown to be useful for energy storage applications such as anode materials for Li-ion batteries [147,148,150]. We have summarised and compared the current state of the art in the vapour-phase self-seeded growth of Ge nanowires in Table 1.

##### Other Vapour-Phase Growth of Self-Seeded Ge Nanowires

The laser ablation technique was first used to synthesis semiconductor nanowires in 1998 [152] and since then has been combined with both CVD and PVD approaches to grow nanowires [153,154,155]. Laser ablation involves irradiating the source material with a laser to generate a vapour flux, which is then carried by an inert gas to a heated substrate. The laser generates a local temperature of up to 5000 °C within few nanoseconds, allowing ablation of the source materials, regardless of their binding energies [156]. While the high temperatures required (>800 °C) limit its scalability for industrial production, this approach is suitable for the rapid growth of nanowires; growth rate in the range of μm/min. Nanowire composition also be easily tuned by varying the source target. Self-seeded crystalline Ge nanowires, coated with a carbon shell, have been obtained using a laser ablation CVD setup [151]. As-obtained Ge nanowires (see Figure 3e) exhibited diameters of between 8 and 35 nm with lengths up to ~1 μm. Covalent bonding between the Ge core and C shell was found to prevent oxidation of the nanowires (see Figure 3f).

Electric arc discharge evaporation techniques are considered very promising approaches for growing semiconductor nanowires. These techniques create concentrated atomic flows of the precursor, resulting in high yields of nanowires that can often be structural tuned. The approach is based on a two-electrode setup, usually a cathode of carbon or tungsten and an anode of the source material of interest. A mixture of gases is also required to enhance and quench the reaction, as well as a background gas (inert gas) which is ionised to generate a plasma (serving as the reactive point). Synthesising Ge nanostructures via arc discharge presents serious challenges due to Ge low melting and boiling points (938 and 2850 °C, respectively) and a fairly high electrical resistivity at room temperature (40 Ω cm in bulk Ge with less than 10^13^ impurity atoms per cm^3^) [157]. High electrical resistivity creates problems when the electric arc ignition takes place, which is not suitable for the transmission of large currents [158,159]. However, promising results have recently been achieved for the growth 1D Ge nanostructures without the involvement of any foreign metal catalysts. Catalyst-free Ge structures with a mean diameter in the order of 1 μm and lengths from 100 μm to 1 mm was achieved via arc-discharge CVD [160]. Even though some tapering of the nanowires was detected, the nanowires presented a smooth surface with the presence of small Ge nanocrystal spheres at their ends. The crystallinity of these nanowires was not confirmed through structural characterisation.

**Table 1:** Summary of main publications of vapour phase-grown self-seeded Ge nanowires. (L: length, d: diameter).

Growth Method	Growth Temperature	Precursor	Morphology	Reference
Oblique angle deposition	330 °C	Ge	Irregular morphology	[134]
X-ray-assisted CVD	300 °C	GeH_4_	L: up to 20 μm	[125]
CVD	490 °C	(GeMe_3_)_2_	d: 5–20 nm L: up to 20 μm	[141]
CVD	365 °C	(SiMe_3_)_3_GeH	d: up to 100 nm (core and shell)	[142]
Thermal evaporation CVD	1050 °C	Ge powder	d: 20–200 nm L: up to 5 μm	[146]
Thermal evaporation CVD	1100 °C	GeO_2_	d: 20–120 nm L: 0.5–10 μm	[149]
Laser ablation CVD	-	Ge-graphite composite	d: 8–35 nm L: up to 1 μm	[151]
Electric arc discharge	-	Ge	d: 1 μm L: 100 μm–1 mm	[160]

#### 3.1.2. The Solution-Phase Growth of Self-Seeded Ge Nanowires

Nanowire growth can also take place in liquid medium. Solution-phase growth methods usually guarantee a high production yield of nanowires, while exhibiting less control over the nanowire growth process compared to easily tuneable CVD methods [11,161]. In this section we have also included a discussion on the synthesis of Ge nanowires in supercritical fluid (SCF) solvents.

SCF synthesis of nanowires was first reported in 1993 [162] and, even though this method requires a pressurised system and suitable reaction vessels, it can be considered a fast growth technique [156]. In this approach, the generation of a liquid-vapour coexistence state depends on the pressure applied (or self-induced by the system), as well as the supercritical temperature of the organic solvent (usually solvents with <300 °C boiling points) [163]. SCFs are interesting growth media as they offer a wider reaction temperature range than usual traditional solution-based techniques [164]. The ability to tune both temperature and pressure parameters can be utilised to promote the solubility, mobility (high diffusivity) and reactivity of precursors; by governing solvent properties such as viscosity, polarity, dielectric constant and heat capacity [165]. The interactions of the solvent with the precursors, intermediates, and by-products can also profoundly modify the reaction equilibrium and kinetics [97].

**Figure 4:**
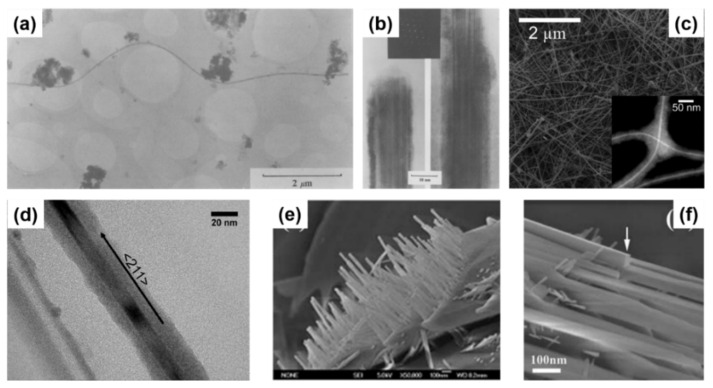
(**a**) TEM image of a 10 μm long, 18 nm diameter, single-crystal Ge nanowire. The islands of material scattered throughout the micrograph consist primarily of bunches of Ge nanodots. (**b**) TEM image of the tip (left) and a central portion of an 18 nm diameter, 650 nm long Ge nanowire. At top left is an ED pattern of the wire, indicating that the wire is a diamond lattice single-crystal oriented along its [011] zone axis. Visible in the micrographs are a set of {200} and {111} lattice planes, running diagonally across the diameter, and a second set of {111} planes, running parallel to the wire axis. Note the faults which traverse the length of the wire. (Parts (**a**,**b**) are reprinted with permission from ref. [162]. Copyright 1993 Elsevier B.V. (**c**) SEM images of the Ge nanowires. Inset is a HAADF STEM micrograph of Ge nanowires synthesised at 400 °C, clearly displaying the core-shell morphology. Including shell, the nanowires shown are 20–50 nm in diameter, while the cores are 8–10 nm in diameter and show good uniformity along the wires. (**d**) TEM image of a nanowire synthesised at 500 °C, showing the distinct core-shell morphology. The image was obtained along the 〈111〉 zone axis of the Ge nanowire. ((**c**,**d**) are reprinted with permission from ref. [166]. Copyright 2010 American Chemical Society. (**e**) Field-emission SEM images of Ge nanowires arrays and (**f**) the SEM of a sectional view of Ge nanowires. (Reprinted with permission from ref. [165]. Copyright 2010 Royal Society of Chemistry).

A great advantage of solution-based techniques is their continuous nature which allows them to be scale-up for industrial purposes. The first report of self-seeded growth of Ge nanowires by a solvent-mediated process was back in 1993, using a SCF setup [162]. Heath and LeGoues [162] reported the high-pressure reduction of GeCl_4_ and phenyl-GeCl_3_ in hexane at 275 °C over a time period of between 2 and 8 days, in a batch setup. The diamond cubic (dc) structure of the Ge nanowires was confirmed by XRD and FTIR (Fourier-Transform Infrared Spectroscopy) analysis showed the surface of the nanowires to be terminated with hydrocarbons, oxygen and chlorine. Although the yield of nanowires was low (between 5 and 10%), TEM imaging (see Figure 4a) showed nanowires with diameters between 7 and 30 nm and lengths up to 10 μm. Self-seeded Ge nanowires (see Figure 4b) exhibited a high density of stacking faults parallel to the crystal growth direction, (111) plane in this case, and the faults were present along the entire lengths of the nanowires. The same authors also used a self-tailored Ge precursor to hinder aggregation and coarsening of Ge nanoparticle during the self-seed growth of nanowires [166]. Hexakis (trimethylsilyl) digermane, Ge_2_(TMS)_6_, was used (dissolved in toluene) with a continuous-flow setup at growth temperatures of between 300 and 500 °C and a pressure of 17 MPa. Seedless sub-10 nm Ge nanowires of excellent quality were obtained with a characteristic core-shell morphology (see Figure 4c). The crystalline cores of the nanowires consisted of pure dc-Ge with diameters ranging from between 6.3 and 9.4 nm (depending on the growth temperature) and the shell was found to be mostly an amorphous matrix largely composed of Si and C with some polycrystalline Ge nanoparticles (see Figure 4d). While the amorphous shell has non-uniform thickness, the core diameter remained uniform along the length of the nanowires. The relationship between reaction temperature and diameter distribution was also studied. In particular, a model was developed to describe the nanowire formation stages (nucleation, coalescence and Ostwald ripening) to explain the influence of growth temperature on the diameter of the seedless Ge nanowires produced [167].

Hydrothermal synthesis is a specific variant of the SCF technique, where the solvent used is supercritical H_2_O. The technique is very flexible and offers a unique scenario to preparing high-quality Ge nanowires at relatively lower temperatures using a simple and cost-effective process [168]. Another advantage is the use of a non-toxic and eco-friendly solvent, H_2_O, in contrast with the other solution-based techniques [169,170]. Hydrothermal techniques have brought excellent results in the self-seeded growth of Ge nanowires. In a simple batch setup using Ge powder and distilled water in a reaction kettle, crystalline Ge nanowires were produced after 6 h at temperatures between 450 and 470 °C under stirring [165]. The nanowires produced displayed smooth surfaces and straight morphologies, with no spherical seeds at the tips of the nanowires (see Figure 4e). TEM observations of the nanowires suggested a rectangular cross-section (see Figure 4f) with a mean diameter of 50 nm (ranging from 10 to 150 nm) and a single-crystal structure of tetragonal germanium (ST12-Ge), although this particular crystallinity was only confirmed by Transmission Electron Microscopy (TEM) and no other characterisation techniques, e.g., Raman spectroscopy or XRD, were used to corroborative the result. Such an interesting outcome in terms of achieving a novel metastable phase of Ge is of high interest and a more detailed characterisation would have been required to verify the crystal structure and phase of these nanowires.

Another high-yield nanowire production method is ‘solution-phase synthesis’ [11]. Here, the growth environment is not a supercritical fluid, rather an organic solvent at ambient pressure. This technique probably represents one of the most simple methods for growing nanowires, as nanostructures can be produced without the need for complex and high-cost equipment or high vacuum systems [75,171]. Growth of Ge nanowires in the liquid phase is usually conducted in high-boiling-point (HBP) solvents, with boiling points typically above 400 °C, e.g., squalane and squalene [172]. This group of organic solvents permits Ge nucleation in a non-pressurised system, facilitating Ge precursor decomposition and nanowire growth [173,174]. A large number of studies on the solution-phase synthesis of Ge nanowire have focussed on self-seeded approaches for nanowire growth [166,175]. A colloidal synthetic approach has been used for the growth of nanocrystals and nanorods in HBP solvents. This approach has also profited from the use of surfactants to grow 1D nanostructures [176]. In this case, surfactants are used to selectively passivate certain facets of the evolving nanocrystal to allow growth along a certain direction, forming nanowires. The solution-phase growth of Ge generally requires reaction temperatures to be high enough to thermally decompose Ge organometallic precursors and to successfully nucleate Ge under these growth conditions.

**Figure 5:**
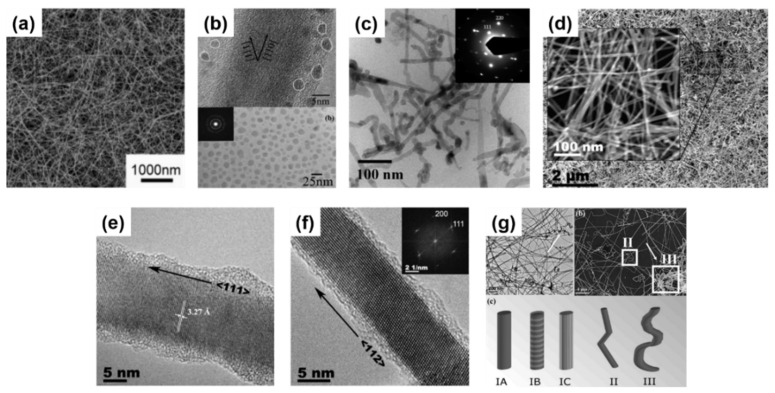
(**a**) SEM image of C–Ge nanowires produced by thermal decomposition of 1.8 × 10^−4^ mole of TOG at 360 °C for 4 h. (**b**) Upper image: HRTEM image showing amorphous-like Ge nanoparticles (marked by circles) with diameters below 7 nm on the surface of one C–Ge nanowire after refluxing for 4 h. Lower image: TEM image for C–Ge nanoparticles with sizes between 10 and 15 nm from the solidification of larger L-Ge droplets in the sample after 1 h refluxing. The inset selected-area electron diffraction (SAED) shows the diamond-type (cubic-structured) Ge. (Reprinted with permission from ref. [75]. Copyright 2007 American Chemical Society). (**c**) Ge nanowires (inset, typical SAED pattern). (Reprinted with permission from ref. [171]. Copyright 2006 American Chemical Society). (**d**) SEM image of nanowires with magnified image inset. (**e**,**f**) HRTEM images of nanowires exhibiting (111) and (112) growth directions, respectively. The interplanar spacing of 3.27 angstroms for the (111) nanowire is shown in (**e**) while an indexed FFT is shown in (**f**). (Reprinted with permission from ref. [172]. Copyright 2011 Royal Society of Chemistry). (**g**) Top-left TEM image of straight Ge nanowires synthesised using squalene as the HBS. Most of the nanowires can be seen to be straight and untapered, while the arrow highlights a small area of kinked nanowires. Top-right SEM image of increasingly kinked nanowires synthesised in squalane with specific nanowire types highlighted. The schematic at the bottom depicts the five different types of nanowires discussed within the text; IA: straight, defect free nanowires, IB: laterally faulted nanowires, IC: longitudinally faulted nanowires, II: angular nanowires and III: more complex, wormlike kinked nanowires. (Reprinted with permission from ref. [177]. Copyright 2011 American Chemical Society).

Zaitseva et al. [175] presented a detail study on the influence of reaction pressure and solvent type on the growth of self-seeded Ge nanowires. Ge nanowire formation from the Ge precursor tetraethylgermane (TEG) at a temperature of ~400 °C and pressure of ≤5 MPa was compared to reactions performed under vacuum and ambient pressure conditions. In both scenarios, HBP solvents, i.e., trioctylamine and squalene and low-boiling-point (LBP) solvents, i.e., hexane and toluene, were screened. The best yields of Ge nanowires, up to nearly 100%, were obtained when reactions took place under pressure in HBP solvents. The use of LBP solvents led to substantially lower yields of Ge nanowires which were mixed with isometric amorphous particles. LBP solvents were not able to produce Ge nanowires in reasonable yields at ambient pressure, at least with the TEG precursor. The choice of self-tailoring a Ge precursor to achieve the desired reactivity under certain reaction conditions has also been explored using different nanowire synthesis techniques. Ge et al. [75] reported an effective Ge precursor for growing highly crystalline Ge nanowires known as TOG, with the molecular formula [(CH_3_(CH_2_)_7_CH = CH(CH_2_)_7_CH_2_NH_2_)_4_Ge]^4+^·(Cl^−^)_4_. These self-seeded nanowires were produced in a three-neck round-bottom flask at a reaction temperature of 360 °C over a time period of 4 h using tri-*n*-octylamine as the solvent. Straight and slightly curved Ge nanowires with diameters between 50 and 70 nm and lengths between 10 and 20 μm were observed (see Figure 5a). A direct correlation between the amount of precursor used and the mean diameter of the nanowires obtained was determined. Amorphous-like Ge nanoparticles, with diameters <7 nm, were observed in the amorphous-carbon coating surrounding the nanowires (see Figure 5b). In another report, Gerung et al. synthesised self-seeded Ge nanowires from a new Ge^2+^ precursor, i.e., Ge(2,6-OC_6_H_3_(C(CH_3_)_3_)_2_) [171]. Although crystalline, the nanowires formed displayed kinked and tapered structures with diameter distributions between 15 and 25 nm and lengths ranging from 100 nm to 10 μm could be found in the samples (see Figure 5c). The use of commercially available Ge precursors have been widely used by several research groups to grow self-seeded Ge nanowires. Barrett et al. [172] used the commercially available precursor diphenylgermane (DPG), under reflux, to grow high yields of long (>10 μm), untapered Ge nanowires with diameters ranging between 7 and 15 nm (see Figure 5d). All of the nanowires synthesised had diamond-cubic crystal structures, with the majority of nanowires displaying a [111] crystal growth direction and about 20% exhibiting a [112] growth orientation (see Figure 5e,f), consistent with previous reports [171]. A more detailed study on the growth of self-seeded Ge nanowires using the DPG precursor in HBP solvents at a temperature of 420 °C was reported by Geaney et al. [177], with an in-depth analysis of the different morphologies obtained (see Figure 5g). Straight, defect free and single-crystal Ge nanowires, with diameters ranging between 7 and 20 nm, were found to constitute the majority of the samples. However, approximately 5% of nanowires presented lateral and longitudinal stacking faults, which originated during the nucleation process. The lateral defects were found to run parallel to the growth direction for <111> oriented nanowires, while the nanowires were the growth occurs in the <112> direction, the defects keep appearing on the (111) type plane to alleviate the stress. Kinked and very abrupt angular kinked (“wormlike”) nanowires, with diameters between 15 and 40 nm, were found at a higher reaction temperature (450 °C) [177]. We have summarised the solution-phase growth of self-seeded Ge nanowires in Table 2.

**Figure 6:**
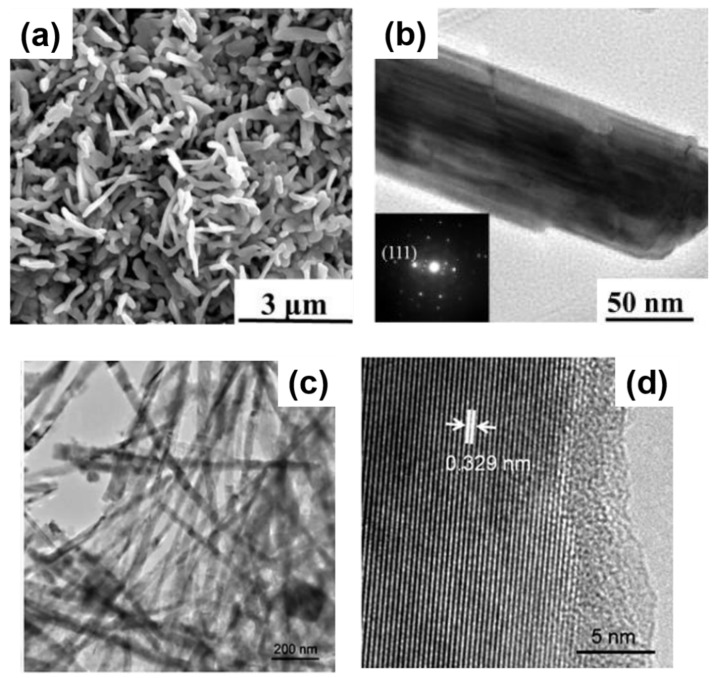
(**a**) SEM images of Ge films obtained from 1.5 M AlCl_3_ + 0.1 MGeCl_4_/[EMIm]Tf_2_N after annealing at 500 °C for 2 h in Ar. (**b**) TEM image and SAED pattern of Ge nanowires. (Parts (**a**,**b**) are reprinted with permission from ref. [52]. Copyright 2017 Royal Society of Chemistry). (**c**,**d**) Regions of darker contrast correspond to elemental Ge while outer walls with lighter contrast are oxide layers of 4 to 8 nm thickness. Surface roughness is clearly observed on the Ge-nanowires in high resolution imaging (**d**). ((**c**,**d**) are reprinted with permission from ref. [183]. Copyright 2011 Royal Society of Chemistry).

Electrochemical deposition of self-seeded nanowires can also be classified as a solvent (electrolyte)-based growth method. Electrochemical deposition is a versatile and cost-effective technique for Ge nanowire production. This technique presents a unique combination of several advantages, such as low operation temperatures (mostly at room temperature), simple setup, ease of scalability (widely present in current large-scale production), tunability and environmental friendliness [178,179,180]. Even though this technique can eliminate the requirements of a catalyst seed, long, kink-free and single-crystal Ge nanowires with the potential to be used in different applications are still a challenge [181]. Chi et al. [182] proposed an electrochemical setup to deposit Ge nanowires onto indium tin oxide (ITO) glass substrates. The unusual morphology obtained (choral-like structures of Ge nanowires) displayed an amorphous structure by TEM observation, although XRD and Raman suggested the formation of a crystalline Ge phase. Subsequently, Hao et al. [52] presented encouraging results with a simple two-step electrochemical method. After elementary Ge nanoparticle formation by ionic liquid electrodeposition, samples were annealed at 500 °C in an Ar atmosphere to generate Ge nanowires. The as-obtained nanowires (see Figure 6a,b) displayed diameters of between 100 and 200 nm and lengths of 2–3 μm, with no tapering or branching, but with a certain degree of kinking and surface roughness.

#### 3.1.3. Templated Growth of Unseeded Ge Nanowires

Templated growth techniques are often used to define the position and growth direction of semiconductor nanowires, without the need for catalytic metal seeds. These techniques have been investigated to grow Ge nanowires in high densities with controlled placement and alignment.

Templated growth of semiconductor nanowires can be achieved by depositing material into nanochannels formed within a substrate or membrane. A commonly used nanoporous membranes for templating nanowires is anodised aluminium oxide (AAO) [184,185]. AAO templates not only allow the formation of vertically aligned arrays of nanowires, but also allows control over nanowire diameters. The best methods for depositing metals and semiconductors in the channels of AAO to form nanowires include electrodeposition [186], CVD [187] and epitaxial growth [187]. Yang and Veinot also successfully formed Ge nanowires by thermally reducing GeO_2_ sol-gels within the pores of AAO membranes [183]. The sol-gel was thermally reduced at 600 °C in an atmosphere of Ar/H_2_ for 5 h to generate Ge nanowires with the intention to minimising carbon contamination [188]. Temperature and time were the two main variables used to control nanowire growth which led to the formation of Ge nanowires with tailorable diameters and lengths, between 30 and 300 nm and 2–7 μm, respectively (see Figure 6c,d) [189]. Sol-gels are formed by condensing a suspension of colloidal particles (the sol) to yield a gel and the technique has evolved into a general and powerful approach for producing a range of nanostructures [190]. Sol-gel synthesis has also been combined with other synthetic steps to produce nanowires. Template-aided synthesis combined with electrophoretic deposition, or reductive thermal processing, has also proved to be efficient for Ge nanowire growth. Key parameters for tuning the properties of sol-gel-derived nanostructures are pH, oxidation state and temperature [191].

## 4. Growth Mechanism for Self-Seeded Ge Nanowires

### 4.1. Nanoparticle Seeds

**Figure 7:**
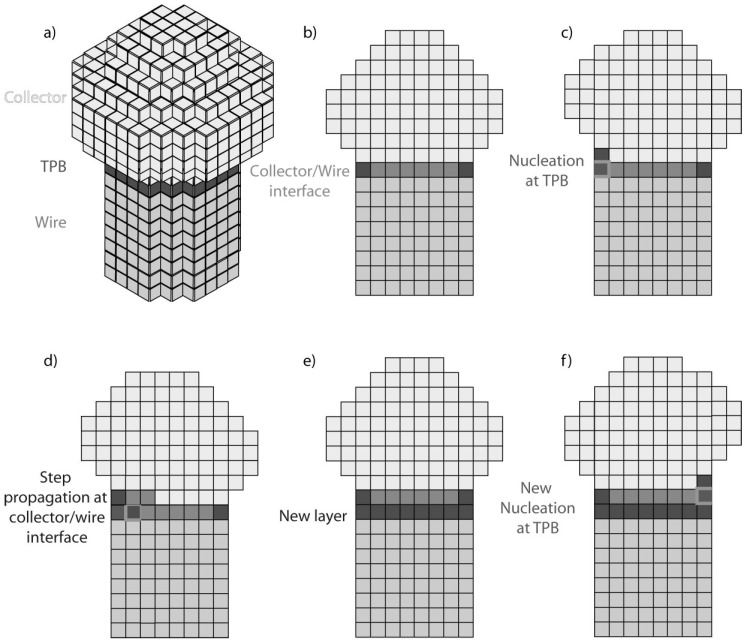
(**a**) An illustration of preferential interface nucleation, birth and spread growth of a nanowire. (**a**) 3D depiction of a wire illustrating the TPB as a dark line on the circumference of the collector/crystal interface. (**b**) 2D cross section of the wire depicted in (**a**). (**c**) Nucleation at the TPB with the TPB being displaced in the growth direction. (**d**) Step propagation at the collector/crystal interface. (**e**) The new layer is completely formed. (**f**) Nucleation at a different site. (Reprinted with permission from ref. [192]. Copyright 2009 Wiley Online Library).

Self-seeded growth of Ge nanowires typically involves the formation of in situ Ge nanoparticle seeds. Similar to the VLS growth of nanowires, the self-seeded growth of semiconductor nanowires involves three components: (i) the precursor dispersed in a medium (supply phase), (ii) the seed particle (collector phase) and (iii) the nanowire (crystalline solid phase) [192]. The presence of the nanoparticles at the tips of nanowires is dependent on a balance between the vapour-nanoparticle, vapour-substrate/nanowire and nanoparticle-substrate/nanowire interfacial energies [193]. These three junctions combine in a singular area commonly known as a three-phase boundary (TPB), and this trijunction is where nanowire growth occurs (see Figure 7). For growth to take place, the first step prior to nucleation of the nanowire is the delivery of molecular or atomic precursors to the seed particle. This transport may happen through vapour, solution or a supercritical fluid phase. This process is known as ‘accommodation’ and can be split in two different steps: (i) adsorption of the precursor adatom onto the seed particle/medium interface and (ii) incorporation of the precursor adatom into the new crystalline solid phase. The incorporation of atoms into a nanowire structure may also occur via diffusion of adatoms along its sidewalls, via a VS mechanism [194]. When the molecule decomposition process to liberate adatoms takes place, both in the nucleating seed or along a nanowire’s sidewall, the organic ligands in the precursors may form volatile by-products. These organic substituent, which might be as simple as a hydrogen atom, may interact with the seed or the nanowire surface promoting a surface passivation which may reduce the energy and the reactivity of the surface [195,196]; surface passivation can significantly mitigate VS interfacial growth [197]. Surfactants can also be used in both solution- and vapour-mediated processes to promote the preferential growth of certain facets [198,199]. By utilising three-phase growth and surfactants, uncommon sidewall facets can become stable, allowing modification of the growth direction. Surface facet development during growth may dictate the axial crystallographic alignment of the wires [200,201]. This is due to the chemical potential reduction of the incorporated adatoms during growth and is correlated with the sidewall facet planes and their surface energies [200,202]. This is especially valid for very small nanowires, where the surface energy of the exposed facets becomes much more relevant and may govern the wire into a particular orientation [200,203].

Axial growth of semiconductor nanowires occurs when the concentration of the reactive species, e.g., Ge atoms, reaches supersaturation in the seed particles. The point of supersaturation largely depends on the thermodynamics of the system, however, kinetic factors such as precursor diffusion and incorporation frequency are also relevant [204]. The generation of crystal building blocks followed by the aggregation of one another to form seed nuclei is generally known as the nucleation step [192]. Growth will then occur when accommodating more precursor in the drop is no longer thermodynamically favourable [192].

A key parameter which significantly influences self-seeded nanowire growth is the diffusion of the seeds (Ge in this case) on the surface of a substrate before the nucleation of the nanowires. This diffusion may promote coarsening and Ostwald ripening effect [205], leading to the formation of bigger nanoparticle seeds and broadening of nanowire diameter distributions [206]. Factors such as adatom diffusion along the sidewalls of nanowires [207] and diffusion and coarsening of the nucleation seed during self-seeded nanowire growth may also lead to tapered nanowires [207]. Other critical factors which can influence crystal structure, the crystal growth direction and the tapering of the nanowires include the wettability and contact angle between the initial seed nanoparticles with the substrate, and later with the nanowire growth face [208,209,210,211,212]. The volume and phase of nanoparticle seeds should also remain unchanged during nanowire growth to minimised crystal defects such as kinking [213,214,215,216,217]. The growth temperature and precursor partial pressure (precursor concentration) are the main variables that can be used to modify parameters such as contact angle, and seed volume in a fixed system [218].

### 4.2. Proposed Growth Mechanism for Self-Seeded Germanium Nanowires

Generally accepted growth mechanisms for Ge nanowires, including self-seeded nanowire, have been described in Section 2.1. A vast consensus can be found on the growth pathway for metal/metalloid seeded Ge nanowires. Thus, when literature refers to any conventional three-phase bottom-up growth, e.g., VLS, VSS, and SFLS, no additional explanation is usually required to justify the growth of the Ge nanowires. However, no standardised mechanism has been found to justify the formation of Ge catalytic seeds and self-seeded Ge nanowire growth. Conventional growth paradigms, e.g., VLS and VSS, can participate to direct 1D growth with the self-formed seeds of a growth material (described in Section 4.1). However, one important criterion is the initial creation of Ge in nanoparticles from which nanowires grow. The self-seeded growth of 1D nanostructures does not involve any foreign metals, metalloid or oxides. The only involvement of third-party material in any self-seeded and unseeded growth of Ge nanowires is for OAG, where oxides, i.e., GeO_x_, are believed to assist in 1D growth. The growth mechanism for OAG has been previously discussed in detail in Section 2.1.

However, in most of the self-seeded growth processes, no third-party catalyst is intentionally added into the reaction. The VS growth mechanism is able to explain some aspects of self-seeded nanowire growth, but it is inadequate or incomplete for most cases. Generally, most of the studies on self-seeded Ge nanowires extensively propose a growth mechanism to explain novel growth behaviour observed in experiments. Thus, it is not possible to represent the self-seeded growth of Ge nanowires by a single growth mechanism. In this section, we will summarise and group all of the suggested growth mechanisms for self-seeded Ge nanowire growth. We will also try to establish a common link between these growth mechanisms in an attempt to standardise self-seeded growth processes.

#### 4.2.1. Growth Mechanisms of Self-Seeded Growth by Vapour-Phase Methods

**Figure 8:**
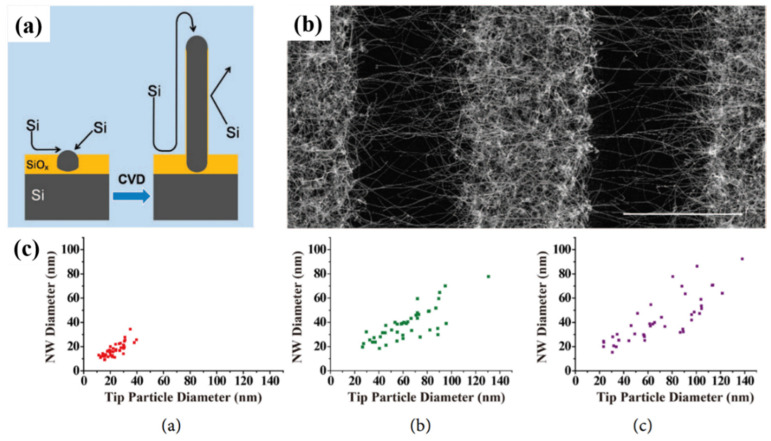
(**a**) A schematic for the proposed catalyst-free Si nanowire growth model. (**b**) A SEM image of metal-free Si nanowires grown on a patterned substrate; scale bar 10 μm. The growth substrate was photolithographically patterned using a silicon (100) wafer with a 50 nm oxide layer. The selected area was chemically etched to remove oxide layers and treated with ultrapure water at 100 °C to generate the reactive SiO_x_ surface. ((**a**,**b**) are reprinted with permission from ref. [138]. Copyright 2009 American Chemical Society). (**c**) Plots of nanowire diameter versus tip nanoparticle diameter obtained at (**c**(**a**)) 0.1; (**c**(**b**)) 0.5; and (**c**(**c**)) 0.9 MPa. (Reprinted with permission from ref. [151]. Copyright 2017 Scientific Research).

Within the group of vapour phase-grown self-seeded Ge nanowires, several mechanisms have been proposed. Among these, classical VS growth was proposed to be mainly responsible for self-seeded growth of Ge nanowires, as reported by Kim et al. [138] in 2009 and Wu et al. [149] in 2010. Kim et al. achieved nanowire growth via a CVD setup using GeH_4_ as a precursor [138]. They did not attribute the self-catalysed nanowire growth to a VLS mechanism [219] but instead proposed that the initial growth seeds that nucleated nanowires, in a thermodynamically preferred direction (see Figure 8a), were derived from etching of the hydrogen-terminated silicon oxide wafer. Higher densities of nanowires were observed on activated regions of the substrate (see Figure 8b). In another report, Wu et al. [149] proposed mixtures of OAG and VS growth mechanisms using GeO_2_ as a precursor for the self-seeded growth of Ge nanowires. In the coldest substrate reaction zone (645 °C), participation of both VS and OAG growth produced Ge nanowires at high densities [93], whereas in the hottest substrate zone (720 °C), nanowires were produced by OAG combined with self-catalytic VLS growth [220]. The nanowires present in the hottest area of the substrate showed a spherical shape on their tips due to the formation of a liquid droplet, which lead to nanowire growth via a self-catalytic VLS mechanism. A similar self-seeded VLS growth mechanism was also observed for the growth of tin oxide nanostructures [221,222].

Demaria et al. [125] used an X-ray-assisted CVD technique to grow self-seeded Ge nanowires. The influence of the X-rays and reaction temperature on nanowire growth was explored. While temperature was essential for the growth of any nanostructure, X-ray were deemed to be a growth enhancer due to a lower yield of nanowires in their absence. Radiolytic activation was believed to accelerate the dehydrogenation of the Ge precursor (GeH_4_ in this case). A thin amorphous layer of Ge was also observed on the substrates which was also assumed to participate in nanowire nucleation. An oversimplification might lead us to conclude that the nuclei formation was caused by naturally activated sites on the substrate (as previously reported by Kim et al. [138]) or oriented heteroepitaxy (as previously reported by Li et al. [134]). However, massive H_2_ formation due to X-ray dehydrogenation and the substantial presence of the H_2_ near the substrate are hypothesised as the reason for nanowires to nucleate. H_2_ interacts with the thin Ge amorphous layer, blocking the surface and leaving few dangling Ge atoms available to act as nuclei to promote the formation of Ge nanowires.

Laser ablation of Ge and C powder can also lead to the formation of Ge nanowires [151]. The presence of spherical shapes at the tips of nanowires and the strong correlations between nanoparticle diameters and nanowire diameters points towards the participation of in situ-formed nanoparticles as a catalytic seeds for nanowire growth (see Figure 8c). The formation of Ge/C liquid droplets was proposed for nanowire growth, analogous to the SiO_x_ nanowire formation from SiO_x_ nanoparticle [223]. Ge and C precipitates from the liquid droplet and a phase separation between Ge and C generate the Ge–C core shell nanowire structures. Similar growth mechanisms were suggested by several other groups for self-seeded Ge nanowire growth [147,148,150]. In these methods, the GeO_2_ precursor was intentionally mixed with a hydrocarbon gas (methane, acetylene and ethanol, respectively) to obtain Ge–C core shell nanowires.

Most of the self-seeded nanowire growths via vapour-phase approaches have been obtained on inert growth substrates, i.e., no chemical or reactive role was played by a substrate. Substrates only accommodate catalytic droplets (Ge nanoparticles in this case) for further nanowire growth. Dřínek et al. [141] screened a series of substrates such as stainless steel, Fe, Mo, Ta, W and SiO_2_ at a reaction temperature of 490 °C, with (GeMe_3_)_2_ as the Ge precursor, to evaluate the role of the substrates in self-seeded Ge nanowire growth. Even though Fe, Mo and Ta form intermetallic phases with Ge, only Fe does it at the reaction temperature of 490 °C, while the others form at temperatures of ~700 and 600 °C, respectively [224]. W and SiO_2_ do not present any intermetallic alloy with Ge [224]. Considering the required necessity of adhesion and limited mobility of the precursor atoms until they anchor at a specific surface site to form a nucleus nanocrystal, Dřínek et al. [141] proposed the physicochemical properties of the substrate surface (such as defects, imperfections and present impurities) as the decisive feature for nanowire formation. However, the presence of the methyl groups during precursor decomposition was not considered. Raman peaks corresponding to amorphous and graphite carbon were found, even though no strong Ge–C interaction was detected by FTIR. The presence of the organic ligands on the substrate may have helped nuclei formation for nanowire growth [151]. In this case, the carbonaceous material might have played an special role in assisting initial droplet stabilisation. The carbonaceous structures on the substrate seem crucial for self-seeded nanowire growth over inert substrates [146]. Wu and Tao proposed a VLS growth mechanism, without any in-depth analysis of the nanowire formation mechanism in the presence of carbonaceous compounds [146]. The presence of spherical tips at the end of the nanorods, which appear to be eutectic droplets, was indicative of a self-seeded VLS process aided by the presence of carbonaceous compounds.

Mathur et al. [225] also explored the growth mechanism of Ge nanowires grown on Fe substrates. No evidence of spherical seeds was found at the tips of the nanowires, unlike those reported by Dřínek et al. [141] for Ge nanowire growth with same Fe substrates. Recently, Dřínek et al. [142] also reported the formation of core-shell Ge-Si/C nanowires in the absence of external catalytic seeds. Additionally, no spherically shaped nanoparticles were observed at the tip of the nanowires, thus a process similar to an OAG mechanism was proposed. Dřínek et al. [142] describe that while starting the reaction form tris(trimethylsilyl)germane, (Si(CH_3_)_3_)_3_GeH, a series of reactions in the vicinity of the substrate surface led to the formation of germane-like structures, with the presence of Si and C. Similar nanostructures were previously formed with very similar precursor in a supercritical setup [166]. The initiation of the growth was triggered by Ge-Si/C droplets formed on high-energy defects, or imperfection sites, on the substrates, which participate to generate seed-free core-shell Ge-Si/C nanowires.

#### 4.2.2. Growth Mechanisms of Self-Seeded Growth by Solution-Phase Methods

A more generalised nanowire growth mechanism has been proposed for self-seeded nanowire growth in solvents compared to vapour. Growth under supercritical conditions is included in this section to simplify the understanding of the rationale. The majority of self-seeded Ge nanowires grown in the solution phase have taken place in organic solvents using metalorganic precursors. Heath and LeGoues presented the first report of self-seeded Ge nanowire growth in 1993 [162]. Taking account of the limited characterisation techniques available at that time for an in-depth analysis of the growth mechanism, various possible explanations were considered by the authors. Essentially, the importance of the organic ligands of the precursors was elucidated. When using GeCl_4_ as a precursor Ge nanoparticle were obtained, while nanowires were only produced using phenyl-GeCl_3_ as a precursor. Phenyl group shows a high tendency to polymerise and in combination with HBP solvents can form catalytic droplets, which act as sinks for Ge atoms, ideal for the self-seeded growth of Ge nanowires.

Zaitseva et al. [175] laid the groundwork by proposing a general growth mechanism for the self-seeded growth of Ge nanowires which was useful in explaining subsequent research on the self-seeded grown of Ge nanowires in solvent phase. They proposed the formation of an organic droplet, analogous to the liquid catalyst seed used in VLS growth, which aids the growth of self-seeded nanowires. When working close to the boiling point of the solvent, small drops of condensed solvent act as a sink for precursor vapour molecules (TEG in this case), similar to the role Au plays in the conventional VLS growth of nanowires [226]. Ge incorporates into the organic droplet, and when saturated, crystallises over a substrate, to initiate nanowire growth. Organic radicals released during the precursor decomposition might also polymerise and catalyse further precursor decomposition [227]. Barrett et al. [172] presented their results with the commercially available Ge precursor DPG in HBP solvents. They proposed a similar mechanism of nanowire growth as described by Zaitseva et al. [175], except they proposed the formation of germane gas due to the decomposition of DPG during the reaction. There is no in-depth study on how different Ge organo-metallic precursors might affect the nanowire growth process, but similar outcomes seem to be obtained using commercially available precursors. The dependence of nanowire diameter on the size of the organic droplets was confirmed by substrate screening, i.e., Pyrex, quartz and ITO [172].

**Figure 9:**
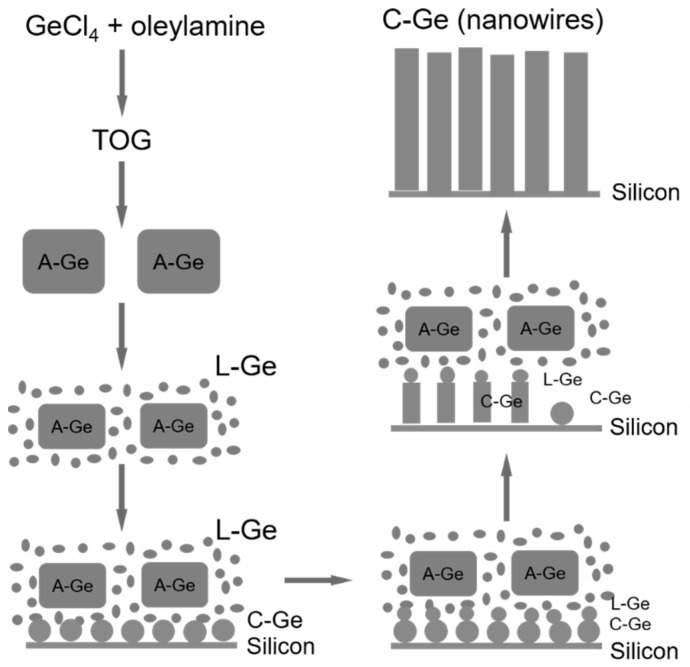
Schematic illustration for the formation of C–Ge nanowires during the decomposition of TOG in TOA solution at 360 °C. (Reprinted with permission from ref. [75]. Copyright 2007 American Chemical Society).

Ge et al. [75] described a new synthetic pathway to explain the growth of Ge nanowires from the self-tailored precursor ([(CH_3_(CH_2_)_7_CH=CH(CH_2_)_7_CH_2_NH_2_)_4_Ge]^4+^(Cl^−^)_4_) (TOG). Decomposition of the precursor (see Figure 9), initiated the formation of liquid Ge droplets on the surface of the substrates. At a critical size, the liquid droplets solidify and act as catalytic seeds for nanowire formation. The growth direction was likely controlled by preferable plane crystallisation, (110) in this case. Although not discussed by the authors, some role may be played by the organic solvent or organic ligands released during precursor decomposition during nanowire growth. Gerung et al. [171] also described the formation of Ge nanowires from the precursor Ge(2,6-OC_6_H_3_(C(CH_3_)_3_)_2_) (germanium 2,6-dibutylphenoxide, Ge(DBP)_2_). They introduced two viable growth mechanisms: (i) a self-seeded VLS mechanism and (ii) a self-assembly mechanism. The first mechanism, agreeing with Zaitseva et al. [175] description, was supported by the presence of a spherical shaped seed at the end of the nanowires which shared a common crystallographic structure with the nanowire. However, the second mechanism was also put forward due to the observance of aggregates of smaller rods near the tip of the nanowires, suggests a continued recrystallisation process of these aggregates extending the length of the nanowires, as also described for Ag [228].

More complex self-seeded Ge nanowire formation mechanisms occur when precursors are used that include Si atoms within the structures. Hobbs et al. [166] obtained core-shell Ge-Si/C nanowires in a supercritical LBP solvent (toluene). The precursor used by Hobbs et al. liberated the Ge atoms prior to the Si species, which immediately formed the initial Ge nuclei [75,229]. The liberated trimethylsilyl groups from the precursor formed a matrix which accommodated molten Ge nanoparticle preventing them from aggregating. These droplets acted as nucleation seeds for Ge nanowire formation. The amorphous shell (composed of Si, Ge, C and O), which passivated the surfaces of the Ge nanowires formed, was not directly involved in the formation of the nanowires beyond nanoparticle stabilisation. This was elucidated by screening different reaction temperatures. Hobbs et al. [166] described in detail some in-between reaction mechanisms to predict the whole reaction pathway for self-seeded nanowire growth. Another interesting growth mechanism for self-seeded Ge nanowires was proposed with H_2_O as solvent, under supercritical conditions [165]. Lin et al. [165] described in detail the reactions of Ge (Ge powder as a precursor) in combination with water ions to nucleate self-seeded Ge nanowires. An analogous method to OAG mechanism was described here with subtle but crucial differences. Here, both H^+^ and OH^-^ ions played an important role, not only on the phase separation but also on the redox reaction in the initial droplet, while phase separation only occurs at the growth tip via OAG mechanism.

## 5. Growth of Self-Seeded Ge Nanowire Alloys

Group IV alloy semiconductor nanowires are attractive due to the ability for bandgap manipulation in these materials, and application in photonics, optoelectronic and nanoelectronics devices. For example, with a Sn concentration of ~9 at.%, GeSn shows a transition to a direct bandgap material from an indirect band gap Ge. Among the reported Ge alloys, such as GeSn [126], SiGe [230], SiGeSn [231,232], only GeSn nanowires have been successfully grown to date through a self-seeded growth mechanism [126]. Adding α-Sn (grey tin, α Sn allotrope with diamond cubic structure) into the Ge lattice results in an energy difference between the Γ and L valleys (ΔE_Γ–L_) that decreases as the Sn content increases, leading to the formation of a direct band gap material at between 6 and 10 at.% Sn content. A key challenge with synthesising GeSn is the low equilibrium solubility of Sn in Ge (<1 at.%) and the tendency for Sn to segregate at high temperatures [233], which can be controlled in nanowires by manipulating growth kinetics.

**Figure 10:**
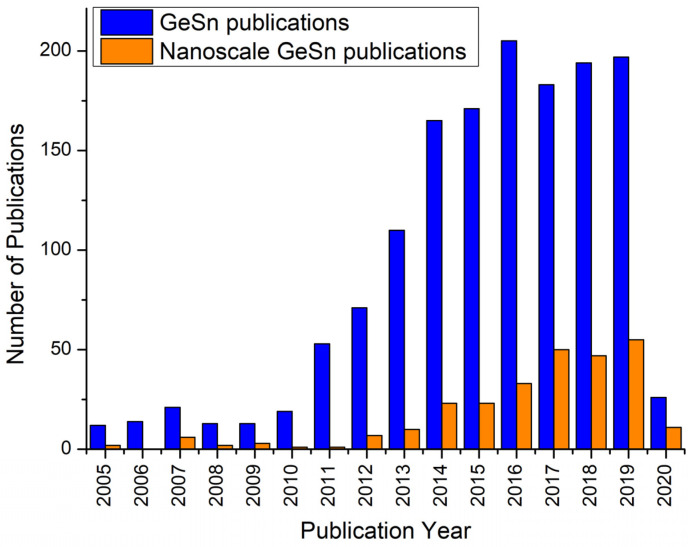
Google Scholar results for publications on GeSn per calendar year. (Reprinted with permission from ref. [234]. Copyright 2020 American Chemical Society).

There are a numbers of recent reports [234] on synthesising GeSn nanomaterials due to their potential application in fields such as electronics [235], optoelectronics [236] and energy storage [237] (see Figure 10). GeSn nanowires have been principally grown to date using pre-synthesised metal catalytic seeds, such as Au [238], AuAg [237], AuSn [239] and Sn [116] nanoparticles. Barth et al. [126] were the first to report the formation of self-seeded GeSn nanowires; formed by heating bis[*N,N*-bis(trimethylsilyl)amido]tin(II) and bis[*N,N*-bis(trimethylsilyl)amido]Ge(II) precursors dispersed in dodecylamine in a microwave. One of the main advantages of microwave heating was that a uniform temperature distribution was achieved throughout the dodecylamine solvent [240,241]. Additionally, GeSn nanowires could be obtained at relatively low reaction temperatures, e.g., 230 °C, within minutes. The GeSn nanowires synthesised displayed a mean diameter of 190 (±30) nm with Sn incorporation of up to 12.4 (±0.7) at.% of Sn. Despite their high Sn content, the GeSn nanowires were crystalline, corresponding to a dc-Ge structure. All of the nanowires synthesised had a bent morphology, which was attributed to the growth kinetics and the solution stirring rate during the growth, which might have created disturbance on the triple-phase boundary at the nanowire growth front [242].

**Figure 11:**
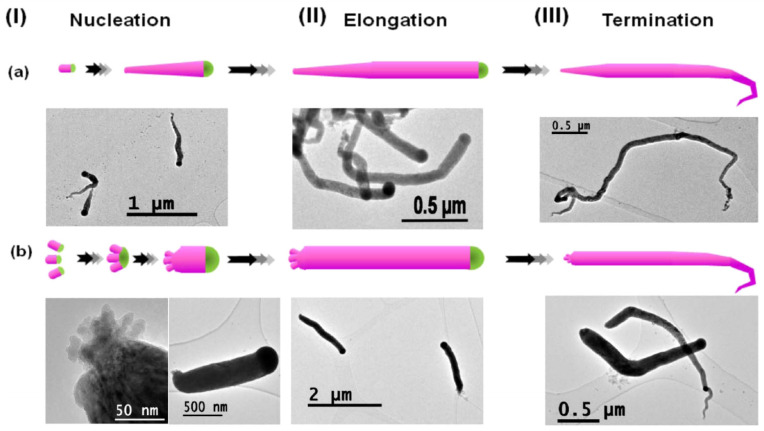
Schematic representation of the three growth stages for Ge_1−x_Sn_x_ nanowire formation and TEM images to illustrate the structural features (Ge in purple; Sn in green). (**a**) Describes the growth via homogeneous nucleation with diameter expansion and accumulation of Sn at the growth front. (**b**) Represents the prenucleation of Ge_1−x_Sn_x_ nanowires by an additional heat treatment and nucleus formation via oriented attachment leading to a quickly settling nanowire diameter at the nucleation (**I**) stage. Elongation (**II**) is a phase where the nanowire grows along its axis with a constant diameter due to constant Sn supply and consumption caused by incorporation in the Ge matrix. Termination (**III**) includes shrinkage in nanowire diameter and the consumption of the tin growth seed. (Reprinted with permission from ref. [243]. Copyright 2015 American Chemical Society).

Seifner et al. [243] reported in-depth analysis of the growth pathway involved in the self-seeded growth of GeSn, as previously described by Barth et al. [126]. They described the in situ formation of different Ge-rich and Sn-rich heterocubanes as the first step in the formation of GeSn nanowires. Those heterocubanes behave as intermediate molecules which would facilitate the growth through a classical SLS mechanism, which is divided into three stages, namely nucleation, elongation and termination (see Figure 11). SLS is usually described through two differentiated steps (nucleation and elongation), although the third extra step in this described growth mechanism was associated with long reaction periods where the Sn-rich seeds at the tips of the nanowires were finally consumed when not enough Sn- and Ge-containing precursor was available. This third step leads to the formation of nanowires with cone-shaped tails, as observed with other growth methods [244]. More recently, Seifner et al. [245] also reported the formation of GeSn at a lower temperature and higher Sn incorporation by using a very similar microwave-assisted growth method. A growth temperature as low as 140 °C was successfully used to obtain GeSn nanorods with Sn content of up to 28 at.%. These globular-shaped GeSn nanorods displayed a diameter distribution ranging between 50 and 250 nm.

## 6. Potential Applications of Self-Seeded Germanium Nanowires

Many potential applications of self-seeded Ge nanowires are related to their semiconductor character, especially in energy storage devices such as Li-ion battery (LIB) anodes. A sub-section dedicated to the potential application of self-seeded Ge nanowires in Li-ion batteries is given below.

### 6.1. Self-Seeded Ge Nanowires in Li-Ion Battery (LIB)

LIBs, compared with metal hydride, lead-acid and alkaline batteries, represent a state-of-the-art energy storage technology [246,247]. However, advancements in commercial LIBs (generally based on LiCoO_2_ and graphite/carbonaceous electrodes) are still required to better their longevity and improve their environmental sustainability, as well as their safety in some applications [248]. Most commercial LIBs employ carbonaceous anodes, which provide low reversible capacities (372 mA h g^−1^ for graphite) due to the formation of LiC_6_, and thus there is a demand for an alternative anode material with a higher energy density and longer life cycles, such as Ge nanowires [249,250,251]. Albeit the high cost of Ge compared to the rest of the group IV elements, the exceptional diffusivity of Li ions in Ge (6.51 × 10^−12^ cm^2^ s^−1^ at room temperature which is 400-fold faster than Si [252], a value estimated from the value obtained by Fuller and Severiens of germanium diffusivity at 360 °C of 2.14 × 10^−7^ cm^2^ s^−1^ by using the empirical equation for the diffusion coefficient) [253], its high theoretical specific capacity (1624 mA h g^−1^) [254], high volumetric capacity (7360 mA h cm^−3^) [254] and high electrical conductivity (100-fold higher than Si) [255] make it a potential material for replacing carbon as an anode material.

Nanowire structures grant efficient electron transport in the axial direction, high interfacial contact and short Li-ion diffusion distances, as well as accommodating electrode volume changes during repeated cycling [12]. Likewise, reducing the diameter of nanowires results in improving electrochemical performance as cracking and pulverisation is significantly reduced [256]. Bottom-up, self-seeded Ge nanowires (Section 3) grown directed onto conductive substrates as a 3D porous mesh of entangled nanowires can act as ideal anodes for LIBs. There is no requirement for binders and/or conductive materials, which add extra weight, inhibit ion transport and degrade the electrical conductivity, unlike with conventional electrodes. However, the challenge remains to determine the critical dimensions and morphologies of nanowires to reduce pulverisation upon repeated cycling of the LIB [257]. The simple fabrication of nanowires is unable to guarantee the structural integrity of Ge anodes, due to the aggregation and large volume changes suffered by the nanowires upon cycling of a LIB. To relieve this volume change, while improving cyclability, several porous materials, such as carbonaceous (amorphous carbon [258], carbon nanotubes [259] and graphene [260]) or oxide coatings [261], have been tested as ‘volume buffer’ materials with Ge-based anodes.

Amorphous carbon has been shown to form as coating on Ge nanowires grown via metal-organic precursor, self-seeded approaches. In LIBs, this amorphous carbon coating can be beneficial due to the formation of a conductive, compact outer-layer on the surface of Ge nanowires. This not only accommodates any volume expansion during cycling but also assists in the formation of a stable solid electrolyte interface (SEI) layer, which is a key factor for the long-time performance of lithium-ion batteries. Additionally, although the final state of Ge lithiation is usually considered to be Li_15_Ge_4_, some authors have pointed to the influence the carbon coating in Ge electrodes has on the formation of Li_22_Ge_5_ [262] or Li_17_G_4_ [263] phases. The appearance of this range of alloys is caused by the state of cycling of the anode materials such as crystallinity, dimensions, and surface/interface conditions [264], and rate and depth of lithiation [264], which modify the kinetics of the different phase transformations into several stable and metastable Li-Ge structures (i.e., Li_7_Ge_2_, Li_9_Ge_4_, Li_15_Ge_4_ or Li_22_Ge_5_) [265,266].

**Figure 12:**
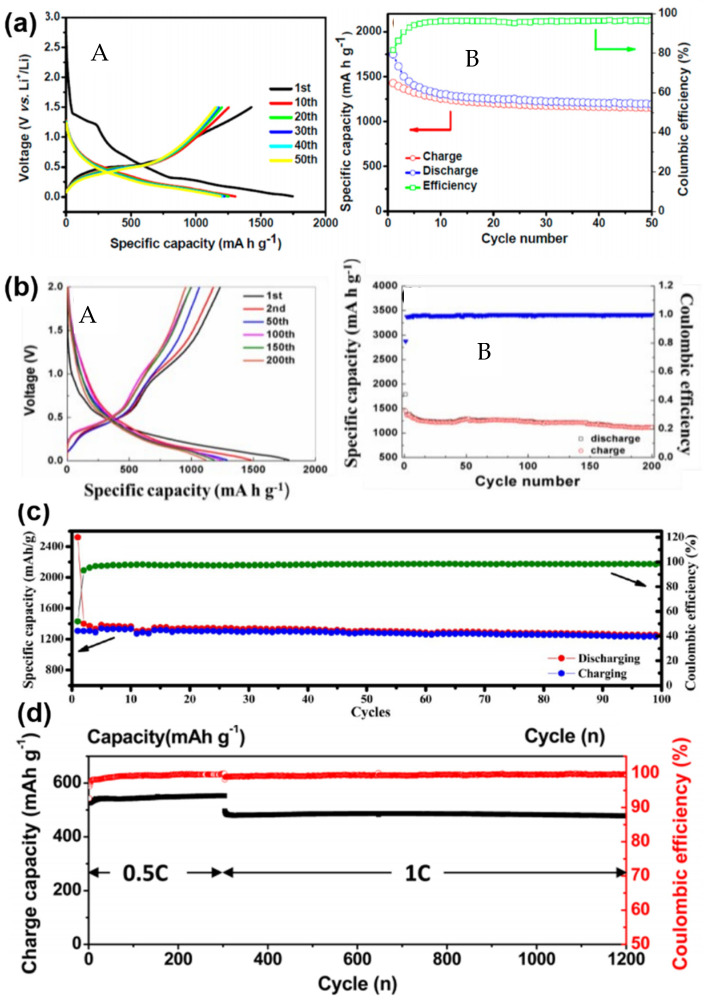
(**a**) Electrochemical performances of Ge/C composite nanowires with full and homogeneous carbon encapsulation. (**a**)-(A) Voltage profiles of Ge/C composite nanowires after 1, 10, 20, 30, 40, and 50 cycles between 0.01 and 1.5 V at a charge/discharge rate of 0.2 C. (**a**)-(B) Plot of specific capacity and Coulombic efficiency of (**a**)-(A) as a function of cycle number. (Reprinted with permission from ref. [267]. Copyright 2014 American Chemical Society). (**b**)-(A) Galvanostatic charge–discharge profiles for different cycles of Ge nanowires at 0.1 C. (**b**)-(B) Cycling performance and Coulombic efficiency of Ge nanowires at 0.1 C. (Reprinted with permission from ref. [52]. Copyright 2017 Royal Society of Chemistry). (**c**) LIB cyclic stability test and rate performance of the germanium-graphite composite, 100 cycles’ capacity measurement. (Reprinted with permission from ref. [150]. Copyright 2015 American Chemical Society). (**d**) Cyclic stability test of the carbon-Ge nanowire electrodes obtained at the 0.5 and 1 C rate. (Reprinted with permission from ref. [147]. Copyright 2016 American Chemical Society).

The most significant data highlighting the positive benefits of using self-seeded Ge/C nanowires in LIBs were obtained by Liu et al. [267], who synthesised GeO_x_/C nanowires and reduced them to Ge/C nanowires under a H_2_ atmosphere at high temperatures to form highly efficient anodes, resulting in a porous Ge nanowire structure anode which was ideal for accommodating Li ion. The specific reversible capacity (calculated only by the mass of pure Ge) obtained at 5 C (Coulomb of electric charge) was 877 mA h g^−1^ (see Figure 12a). Although remarkable capacity retention was observed after 50 cycles, longer testing times would be needed to demonstrate the stability of the electrodes. Hao et al. [52] also tested catalyst-free Ge nanowires for anode in LIBs with similar results. While using a loading density of their electrode of 0.3 mg per cm^2^, the samples showed a capacity of 620 mA h g^−1^ at 5 C (see Figure 12b). An abrupt drop in the charge and discharge capacities identified in the first few cycles was due to SEI layer formation. After 20 cycles, Coulombic efficiency reached values over 98% and good capacity retention was observed after 200 cycles (see Figure 12a,b). Other interesting results using Ge–C nanowires as anodes in LIBs were reported by Sun et al. [150]. In their study, Ge nanowires coated by multilayer-graphite tubes exhibited a reversible capacity of 515 mA h g^−1^ at a current density of 5 C (see Figure 12c). Although stability tests were only undertaken for 100 cycles, great capacity retention was obtained for the nanowires. In another report, Choi et al. [147] presented very poor results for catalyst-free carbon-sheathed Ge nanowires with a specific capacity below 260 mA h g^−1^ at a rate of 5 C (see Figure 12d).

### 6.2. Self-Seeded Ge Nanowires in Other Applications

#### 6.2.1. Self-Seeded Ge Nanowires in Semiconducting Devices

In order to see the effect of metal-free growth on the nanowire performance, Connaughton et al. [268,269] presented two reports analysing the conductivity displayed by core-shell Ge-Si/C nanowires. Two types of nanowires were produced by tuning the growth temperature and reaction time, with comparably contrasting electrical properties. The first type of nanowires were produced via a single-step synthetic process, where the nanowires had a greater presence of Ge nanocrystals in their amorphous shell. Another type of nanowires produced by annealing in a two-step synthetic process had a lower amount of Ge nanocrystals present in their amorphous shells. Both nanowires showed greater electrical conductivity, one order of magnitude higher than the conductivity of bulk Ge [270]. Ge nanowires grown via the single-step process displayed a p-type behaviour with low conductance values, when compared with nanowires grown via the two-step process. The conductance values presented non-linearity and hysteresis, varying at approximately 25 nS, which is indicative of memristance [271,272]. They also exhibited an intrinsic mobility between 10^−3^ and 10^−1^ cm^−2^ V^−1^ s^−1^, compared with the mobility of holes in bulk Ge (1.9 × 10^2^ cm^−2^ V^−1^ s^−1^), which indicates a higher carrier concentration in the nanowires than in bulk Ge [273]. Nanowires grown via the two-step process, with a lower amount of crystalline Ge present in the amorphous shell, displayed linear and higher conductance values, with no field effect present. These nanowires displayed quasi-metallic behaviour compared with degenerately doped Ge (doping density of from 10^19^ cm^−3^ onwards), due to the absence of field effect and the high conductivity displayed [274,275,276]. Nanowires grown via a two-step process displayed resistivity values that rapidly decreased with nanowire diameter, from 40 to 20 nm. This observation was explained by the quantum behaviour of the charge-carriers at small diameters (below Bohr radius ≈24 nm) [37]. The versatile behaviour of the nanowires with slightly different structural features satisfies the requirement of both conduction-channel and source- and drain-components for nanoscale semiconductor devices.

#### 6.2.2. Self-Seeded Ge Nanowires for Field Emission

Ge nanowires could be used as potential field emitters, and some self-seeded Ge nanowires have been screened for this application. Comparison of the emission properties between different Ge nanowire samples is difficult due to their different morphologies, which affects the geometric enhancement factor *β* that describes how electric fields are affected by geometry and surface. Wu et al. [149] reported VS-grown Ge nanowires (diameter of approximately 60 nm) with a turn-on field (applied field to draw an emission current density of 100 nA cm^−2^) of 4.6 V μm^−1^. Li et al. [134], instead, obtained a turn-on voltage of 8.5 V μm^−1^ for the Ge nanowires grown by OAG. The turn-on field improved to 7.6 V μm^−1^ at 1 μA cm^−2^ when the nanowire sample was annealed at a temperature of 550 °C.

There are also a few other innovative applications of self-seeded Ge nanowires. For example, they are used as bio-regenerative and cell proliferation materials, particularly in mammalian cells [277].

## 7. Conclusions and Outlook

The aim of this review was to outline and benchmark synthesis methods and describe growth mechanisms for self-seeded Ge nanowires. Advances reported in the last two decades have drastically enhanced the possibility of application of self-seeded Ge nanowires in a wide variety of fields. Si has been, almost from the very beginning, the material of choice for the microelectronics industry, while graphite (carbon) has played the same role in the LIB industry. Predictions indicate that the dependence on microelectronics and LIBs will continue to expand and new materials will be required to fulfil the performance demanded by consumers. Ge nanowires, in general, present numerous advantages which place them as a genuine alternative in many application fields.

Self-seeded Ge nanowires represent a novel material which can overcome impurity issues associated with metal-catalysed Ge nanowires, while displaying competitive nanowire growth rates, narrow diameter distributions and comparable morphologies. Self-seeded Ge (and Si) nanowires have enormous potential in future nanoelectronics, optoelectronic and energy storage devices. However, there seems to be a lack of recent development on this method and material, which is evident from the low number of recent research articles on the subject. This could be due to the fact that unlike the very popular three-phase seeded growth of Ge nanowires, no systematic growth protocols or trends are developed for the self-seeded growth of germanium (or silicon), as disclosed in this review. This highlights the need to devote attention to these materials and growth methods, considering the significance that self-seeded (or seedless) Ge (or Si) 1D materials can have in future device applications.

This review look into this relevant subject in detail, and summarises progress in the bottom-up growth and growth mechanisms of self-seeded Ge nanowires, also examining the applications fields of greater interest. A considerable number of growth methods were detailed. The various growth methods discussed are quite distinct, and the question of which method is best mainly depends on the application. Most of the approaches described generate meshes of entangled nanowires, which may require a subsequent positioning and assembly step for some applications such as in nanoelectronics. A lack of research for in-place fabrication methods, that is, the synthesis of nanowires at specific positions on a substrate, is still a challenge which needs to be overcome. Success would allow the bottom-up growth approaches to directly compete with the top-down fabrication methods, in terms of controllability, reliability and size variability.

The growth mechanisms behind the self-seeded growth of Ge nanowires still needs to be understood in more detail and this is key to the implication of this method for functional material fabrication. Solution-phase methods share many commonalities and the presence of organic ligands (carbonaceous structures) and their decomposition pathways seem to play a vital role in the growth of self-seeded Ge nanowires. In solution-phase growth, a surfactant or ligand can be added to the reaction. However, the preference should be in situ generation of the templating molecules during nanowire growth to infer the cost-effectiveness of the method. In this regard, in situ polymerisation of reaction by-products in a suitable environment (e.g., SCF atmosphere) could play a key role in template-mediated nanowire growth. The key is to prevent the homogeneous nucleation of spherical aggregates by confining them in a carbon-based polymeric template. This could be externally induced, or better in situ generated.

Vapour-phase methods, however, still show a lack of connection, and deeper exploration and more detailed studies are required. Nevertheless, connections have been found between the different approaches reported in the literature. Though an external template such as AAO is expansively used for seedless growth of Ge nanowires, crystallinity, morphological breakdown, and doping are some of the issues associated with this approach. In situ template generation to confine the radial growth of the nanostructure is also key to the vapour-phase growth of self-seeded nanostructures. Usually, to achieve this, engineered metal-organic precursors are utilised. This makes the vapour-phase growth of self-seeded Ge nanowires complicated and expensive. A future aim should be to develop a method/mechanism to utilise the commercially available precursors for the growth of self-seeded nanowires. Thus, the key to vapour-phase growth of self-seeded or seedless nanowires is to select and design precursors which can form a template during the reaction to confine the Ge nanoparticles for subsequent use as growth seeds for nanowire growth. The growth happens via the nucleation of Ge nanoparticle and subsequent attachment of Ge adatoms at the catalyst (Ge nanoparticle in this case)–nanowire interface via a pseudo VLS-like growth. In another scenario, nucleated Ge nanoparticles can form 1D nanostructures via coalescence and Ostwald ripening inside in situ-generated organic or inorganic templates. The growth mechanism for the self-seeded growth depends on the choice of chemicals, reaction kinetics, thermodynamics and surface energy of the growth system. Along with the localised growth, future objectives should be precise control over the morphology, dimension, crystal structure and surface faceting.

Among all the described applications, energy storage, LIBs in particular, is seen as a promising application area for self-seeded Ge nanowires. This is due to the fact that the nanowires are usually confined within a template during the self-seeded growth, which renders higher structural stability during electrochemical cycling. Structural stability can result in excellent capacity performance and cyclability. Additionally, an amorphous carbon-based template, formed during the self-seeded nanowire growth, can also positively influence specific capacity and capacity retention by drastically enhancing the reaction kinetics during cycles by promoting electron transport, increasing electrical contact points, as well as providing more paths for charge carrier transfer. For nanoelectronics applications, interfacial states between the nanowire and the shell can be controlled via variation of the shell morphology. This, in turn, can control device characteristics such as carrier concentration and mobility. Thus, self-seeded nanowires with in situ-generated shells offers the possibility of controlling the functionality of nanowire devices by simply controlling the interfacial morphology, thus eliminating the need for laborious, cost- and time-intensive doping schemes.

## Figures and Tables

**Table 2:** Summary of main publications of solution phase-grown self-seeded Ge nanowires. (L: length; d: diameter).

Growth Method	Growth Temperature	Precursor	Morphology	Reference
SCF	273 °C	GeCl_4_ and phenyl-GeCl_3_	d: 7–30 nm L: up to 10 μm	[162]
SCF	300–500 °C	Ge_2_(TMS)_6_	d: 6.3–9.4 nm	[166]
Hydrothermal	450–470 °C	Ge powder	d: 10–150 nm	[165]
SLS	~400 °C	TOG	d: <50–700 nm	[175]
SLS	360 °C	TOG	d: 50–70 nm L: 10–20 μm	[75]
SLS	~300 °C	Ge(2,6-OC_6_H_3_(C(CH_3_)_3_)_2_)	d: 15–25 nm L: 100 nm–10 μm	[171]
SLS	415 °C	DPG	d: 7–15 nm L: >10 μm	[172]
SLS	420 °C	DPG	d: 7–20 nm	[177]

## Abbreviations

Full phrase	Abbreviation
Anodised aluminium oxide	AAO
Chemical vapour deposition	CVD
Diphenylgermane	DPG
Fourier-Transform Infrared	FTIR
High boiling point	HBP
Indium tin oxide	ITO
Lithium-ion batteries	LIB
Low boiling point	LBP
Oblique angle deposition	OAD
Oxide-assisted growth	OAG
Physical vapour deposition	PVD
Solid electrolyte interface	SEI
Solution–liquid–solid	SLS
Supercritical fluid	SCF
Supercritical fluid–solution–solid	SFLS
Transmission Electron Microscopy	TEM
Tetraethylgermane	TEG
Three-phase boundary	TPB
Vapour–liquid–solid	VLS
Vapour–solid–solid	VSS
X-ray Diffraction	XRD
